# Electro-osmotic capture and ionic discrimination of peptide and protein biomarkers with FraC nanopores

**DOI:** 10.1038/s41467-017-01006-4

**Published:** 2017-10-16

**Authors:** Gang Huang, Kherim Willems, Misha Soskine, Carsten Wloka, Giovanni Maglia

**Affiliations:** 1 Groningen Biomolecular Sciences & Biotechnology Institute, University of Groningen, 9747 AG Groningen, The Netherlands; 2 KU Leuven Department of Chemistry, Celestijnenlaan 200G, 3001 Leuven, Belgium; 3 Imec, Kapeldreef 75, 3001 Leuven, Belgium

## Abstract

Biological nanopores are nanoscale sensors employed for high-throughput, low-cost, and long read-length DNA sequencing applications. The analysis and sequencing of proteins, however, is complicated by their folded structure and non-uniform charge. Here we show that an electro-osmotic flow through Fragaceatoxin C (FraC) nanopores can be engineered to allow the entry of polypeptides at a fixed potential regardless of the charge composition of the polypeptide. We further use the nanopore currents to discriminate peptide and protein biomarkers from 25 kDa down to 1.2 kDa including polypeptides differing by one amino acid. On the road to nanopore proteomics, our findings represent a rationale for amino-acid analysis of folded and unfolded polypeptides with nanopores.

## Abstract (web summary)

Biological nanopore–based protein sequencing and recognition is challenging due to the folded structure or non-uniform charge of peptides. Here the authors show that engineered FraC nanopores can overcome these problems and recognize biomarkers in the form of oligopeptides, polypeptides and folded proteins.

## Introduction

In nanopore biopolymer analysis, molecules are recognized by the characteristic modulation of the ionic current during their residence inside the nanopore under an external potential. Nanopore sensors are advantageous because they recognize single molecules and the ionic signal can easily be interfaced with low-cost and portable electronic devices. Most notably nanopores can be used for nucleic acid analysis^1–7^ and sequencing^8–13^ where individual DNA strands are unfolded and stretched by the electric field as they are fed through the nanopore. The analysis of proteins and polypeptides, however, is complicated by the fact that they do not possess a uniform charge distribution. Depending on its direction, the electrical field can either facilitate or retard the transport of charged residues, or have almost no net contribution to the transport of neutral amino acids. Therefore, the translocation and stretching of a polypeptide through a nanopore must be induced by other means, for example, by using enzymes^14^.

Previous work with biological nanopores mainly focussed on the electrophoretic-driven translocation of model peptides^15–19^. More recently, it has been acknowledged that the electro-osmotic flow (EOF), induced by the fixed charges in the inner wall of the nanopore, has considerable influence on the transport mechanism of molecules across nanopores^20–27^. In particular, it was shown that at pH 2.8 positively charged peptides can be trapped inside a nanopore by the balancing effect of the electrophoretic driving force and the opposing EOF through the nanopore^27^. This suggests that the intensity of the EOF can be as strong as the electrophoretic force and, in turn, the EOF might be used to translocate and stretch polypeptides for protein sequencing applications. However, changing the pH of the solution also influences the charge of the nanopore inner surface, and hence the EOF. When using biological nanopores this is an issue, since altering the pH to uniformly charge a polypeptide would also adversely affect the direction of the EOF. For example, at pH 2.8, the inner surface of a biological nanopore consisting of natural amino acids would be positively charged, resulting in an EOF from *cis* to *trans* under positive applied voltages at the *trans* side. The positive applied potential, however, opposes the translocation of the protonated polypeptides.

Folded proteins can also be characterized with nanopores. Biological nanopores such as OmpG^28, 29^, αHL^14, 30^, phi29 DNA-packaging motor^31^, and FhuA^32^ have been used to recognize proteins interacting at the entry of the nanopore. More recently, we have shown that folded proteins can be sampled using the larger ClyA nanopore, where the EOF traps proteins with a certain size inside the ~6 × 10 nm nanochamber of the nanopores^23, 33–36^. Inside the nanopore, proteins remain folded and the ionic current can distinguish between different proteins^23^, isomeric DNA:protein binding configurations^34^, and ligand-induced conformational changes^36^. Work with solid-state nanopores with a large diameter revealed that globular proteins might translocate too quickly across nanopores to be properly sampled^37^. However, if the diffusion of the protein is controlled by modulation of pH^38^, immobilization within the nanopore’s walls^39, 40^, or by the interaction between a protein and the nanopore^41–43^, ionic current blockades can be used to identify proteins.

The shape, size, and surface charge of the nanopore are important factors for the recognition of proteins. Proteins with different masses have been studied with different size glass and solid-state nanopores. The groups of Keyser^44^ and Radenovic^45^ used glass nanopores with diameters of >20 nm for the analysis of proteins ranging from 12 to 480 kD relying on the electrophoretic force for protein capture. Wanunu and co-workers^46, 47^ fabricated smaller solid-state pores (~5 nm diameter) to separate sub-30 kD proteins (28.9 kD ProtK and 13.7 kD RNaseA) and found that the osmotic flow dominated the transport for most of the proteins analyzed. Meller’s group^38^ used even a smaller solid-state pore (3 nm diameter) to sample ubiquitin (8.5 kD) and achieved the separation of ubiquitin chains of different lengths including ubiquitin dimers (~17 kD) of two different conformations. Recently, we showed also the real-time ubiquitination of a model protein with ClyA nanopore^48^. However, the detection and separation of peptides and small proteins are still very challenging and has not been achieved to our knowledge. Moreover, the charge and EOF need to be more carefully tuned for the capture and transport of sub-10 kD polypeptides, complicating the establishment of general conditions for the detection of such small analytes.

**Fig. 1:**
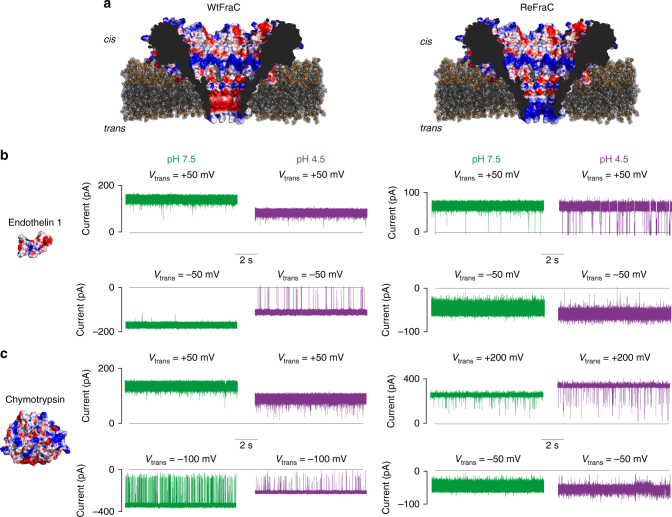
Capture of endothelin 1 and chymotrypsin with two FraC variants at two different pH conditions. **a** Cross sections of wild-type FraC (WtFraC, PDB: 4TSY) and D10R-K159E-FraC (ReFraC). **b**, **c** Representative traces induced by 1 µM endothelin 1 (**b**) and 200 nM chymotrypsin (**c**) to WtFraC (left) and ReFraC (right). Chymotrypsin (PDB: 5CHA) and human endothelin 1 (PDB: 1EDN) are shown as surface representations. Endothelin 1 and chymotrypsin enter WtFraC under negative applied potentials, while they enter ReFraC under positive applied potentials. Chymotrypsin blockades to WtFraC were also observed under −50 mV at pH 7.5 and 4.5; however, the applied potential was increased to −100 mV to obtain a sufficient number of blockades. At pH 7.5, blockades to ReFraC by chymotrypsin under positive applied bias required higher potential than to WtFraC under negative applied bias. The buffer at pH 7.5 included 1 M KCl, 15 mM Tris base, and the buffer at pH 4.5 contained 1 M KCl, 0.1 M citric acid, 180 mM Tris base. Endothelin 1 and chymotrypsin were added into *cis* compartment. All traces were recorded using 50 kHz sampling rate and a 10 kHz low-pass Bessel filter. The coloring represents the electrostatic potential of the molecular surface as calculated by APBS^75^ (pH 7.5 in 1 M KCl) with red and blue corresponding to negative and positive potentials (range from −4 to +4 *k*_B_ *T*/*e*_*c*_), respectively. Structures were rendered using PyMOL

Recently we characterized an α-helical pore-forming toxin from an actinoporin protein family Fragaceatoxin C (FraC) for DNA analysis^49^. The crystal structure revealed that FraC consists of eight small subunits that describe a basket-shaped nanopore with a large opening of ~6 nm diameter at the *cis* entry. The transmembrane region of FraC is formed by eight V-shaped α-helices that taper down toward a narrow constriction of ~1.5 nm at the *trans* entry of the pore (Fig. 1a)^50^. Thus, the narrow constriction of FraC appears ideally suited for protein-sequencing applications, while the large vestibule described by the *cis* lumen of the nanopore might be ideal to characterize small folded proteins. In this work, we use FraC nanopores to recognize biomarkers in the form of oligopeptides (≤10 amino acids), polypeptides (>10 amino acids), and folded proteins (>50 amino acids). We find that the precise tuning of the charges present in the constriction of the nanopore is important to allow the translocation of oppositely charged polypeptides through FraC nanopores. Once inside the nanopore, polypeptides could be identified by their ionic current blockades, suggesting that this technology can be suitable for the proteomic characterization of biological samples.

## Results

### Protein capture with FraC nanopores

To assess FraC nanopores as a sensor for peptide and protein biomarkers, we initially selected endothelin 1, a 2.5 kD polypeptide of 21 amino acids and α-II-chymotrypsin (henceforth chymotrypsin), a 25 kD globular protein of 245 amino acids (Fig. 1). Analytes were added to the *cis* side of wild-type FraC (WtFraC) nanopores (Fig. 1a) containing a 1 M KCl, 15 mM Tris base, pH 7.5 solution, and an external potential was applied to the “working” electrode located in the *trans* compartment. Because WtFraC shows gating above approximately +50 mV, but is stable at potentials as high as −300 mV, a potential at which the lipid bilayer consisting of 1,2-diphytanoyl−sn-glycero-3-phosphocholine becomes presumably unstable, we applied potentials between those limits. Addition of 1 µM of endothelin 1 to the *cis* compartment did not provoke blockades at +50 mV (Fig. 1b) and up to −300 mV. Since the constriction of WtFraC is lined with aspartic acid residues (Fig. 1a), we reasoned that the protonation of these residues at more acidic pH values should diminish the energy barrier for the translocation of endothelin 1, which carries a net charge of −2 at pH 7. Simultaneously, a less negative endothelin 1 would also migrate more easily toward the *trans* electrode under negative applied potentials. Endothelin 1 blockades started to appear at pH 6.4 at –50 mV (Supplementary Fig. 1a), and their capture frequency increased linearly with decreasing the pH (Supplementary Fig. 1b). At pH 4.5 (1 M KCl, 0.1 M citric acid, 180 mM Tris base), endothelin 1 blockades to WtFraC were observed at −50 mV, but not at +50 mV (Table 1; Fig. 1b).

**Table 1:** Parameters of endothelin 1 and chymotrypsin captured with WtFraC and ReFraC

	WtFraC					ReFraC			
	pH 7.5		pH 4.5			pH 7.5		pH 4.5	
	*τ*_on_ (ms)	*τ*_off_ (ms)	*τ*_on_ (ms)	*τ*_off_ (ms)		*τ*_on_ (ms)	*τ*_off_ (ms)	*τ*_on_ (ms)	*τ*_off_ (ms)
Endothelin 1 (−50 mV)	–	–	5.8 ± 0.7	5.6 ± 2.0	Endothelin 1 (+50 mV)	1413.7 ± 286.9	3.3 ± 2.2	401.7 ± 79.4	8.5 ± 1.8
Chymotrypsin (−100 mV)	4.4 ± 1.9	12.0 ± 5.7	9.6 ± 2.5	1.7 ± 0.9	Chymotrypsin (+200 mV)	174.3 ± 22.9	0.2 ± 0.1	112.5 ± 9.5	1.3 ± 0.7

The buffer at pH 7.5 consisted of 1 M KCl, 15 mM Tris base. The buffer at pH 4.5 consisted of 1 M KCl, 0.1 M citric acid, 180 mM Tris base. Endothelin 1 and chymotrypsin were added into *cis* compartment. Recordings were performed using a 50 kHz sampling rate with a 10 kHz low-pass Bessel filter. The errors represent the standard deviations calculated from three experiments

*τ*_*on*_ inter-event time, *τ*_*off*_ dwell time

Encouraged by the effect of a more positive constriction under acidic conditions, we next investigated the capture of endothelin 1 with the D10R, K159E FraC (ReFraC) nanopore, a pore with arginine residues at its constriction, which we previously engineered for purposes of DNA analysis^49^. Conversely to WtFraC, ReFraC is stable under positive applied potentials but displays gating at potentials of approximately −50 mV. Consequently, we only applied voltages between −50 and +200 mV to ReFraC. Addition of 1 µM endothelin 1 to the *cis* compartment elicited blockades at pH 7.5 at +50 mV but not −50 mV (Table 1; Fig. 1b). Decreasing the pH to 4.5 (1 M KCl, 0.1 M citric acid, 180 mM Tris base) led to an increase in capture frequency at +50 mV (Table 1; Fig. 1b), despite the reduced electrophoretic mobility toward the *trans* electrode.

Next we tested chymotrypsin (25 KDa, pI = 8.75) as representative of a relatively large protein analyte. Protein blockades were only observed at negative applied potentials from −50 mV (Supplementary Fig. 2) and higher potentials in pH 7.5 buffer (1 M KCl, 15 mM Tris base). The residual current became homogeneous when we increased the potential to −100 mV (Table 1; Fig. 1c). Contrary to what we observed for endothelin 1, the capture frequency of chymotrypsin remained constant between pH 7.5 and 5.5, and decreased by ~50% when the pH was lowered to 4.4 (Supplementary Fig. 3). Using ReFraC at pH 7.5, we noticed only few blockades at high positive applied potentials but not at −50 mV (Table 1; Fig. 1c). Decreasing the pH to 4.5 led to an increase in capture frequency (Table 1; Fig. 1c). Notably, ReFraC showed often shallow gating events at negative applied potentials under acidic conditions as shown in Fig. 1c, bottom right. Taken together, both nanopores can capture analytes differing 10-fold in molecular weight (2.5 vs. 25 kDa).

### The charge of the constriction dictates the ion selectivity

**Fig. 2:**
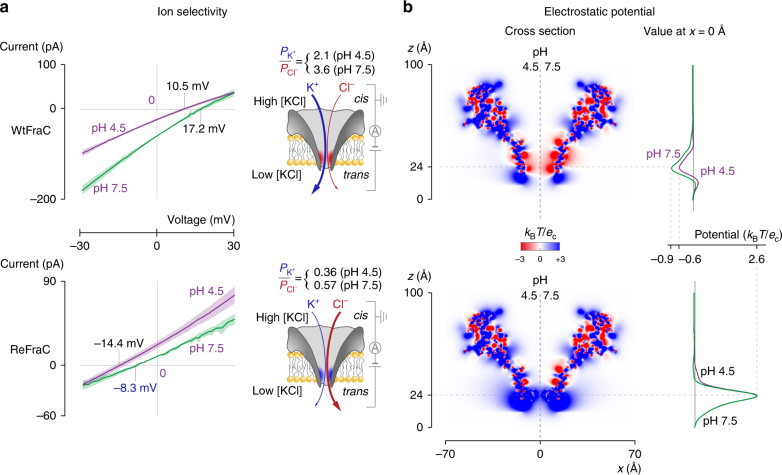
Ion selectivity and electrostatic distribution of WtFraC and ReFraC. **a** Determination of the reversal potential shows that WtFraC and ReFraC are, respectively, cation- and anion-selective, as expected from the electrostatic potentials at their constrictions. All reversal potentials were measured under asymmetric salt conditions (467 mM KCl in *trans* and 1960 mM KCl in *cis*) and the ion selectivity determined using the Goldman–Hodgkin–Katz equation (Eq. 1 in the main text). The buffer contained 15 mM Tris base at pH 7.5 and 100 mM sodium citrate at pH 4.5. **b** The averaged simulated electrostatic potentials reveal the negatively and positively charged constrictions of WtFraC and ReFraC, respectively. While for ReFraC lowering of the pH from 7.5 to 4.5 only had a small effect on the electrostatic potential, for WtFraC the peak value at the center of the constriction dropped by 37%. All simulations were performed using APBS^75^ at 1 M KCl, with the partial charge of each titratable residue adjusted according to their average protonation states with a modified version of the PDB2PQR software^72^. Residue p*K*a values were estimated using PROPKA^78, 79^. Detailed experiment procedures are given in Methods section. The envelopes behind every current–voltage curve represent their respective standard deviations obtained from three repeats

To collect details of the ion transport across WtFraC and ReFraC pores, we measured the ion selectivity of WtFraC and ReFraC pores using asymmetric KCl concentrations on either side of the nanopore (1960 and 467 mM). The reversal potential (*V*_r_), i.e., the potential at which the current is zero (Fig. 2a), was then used, together with the Goldman–Hodgkin–Katz equation, to calculate the ion selectivity (${P_{{{\rm{K}}^ + }}}/{P_{{\rm{C}}{{\rm{l}}^ - }}}$) of both nanopores:

1 $$\frac{{{P_{{{\rm{K}}^ + }}}}}{{{P_{{\rm{C}}{{\rm{l}}^ - }}}}} = \frac{{{{\left[ {{a_{{\rm{C}}{{\rm{l}}^ - }}}} \right]}_{{\rm{trans}}}} - {{\left[ {{a_{{\rm{C}}{{\rm{l}}^ - }}}} \right]}_{{\rm{cis}}}}{e^{{V_{\rm{r}}}F/RT}}}}{{{{\left[ {{a_{{{\rm{K}}^ + }}}} \right]}_{{\rm{trans}}}}{e^{{V_{\rm{r}}}F/RT}} - {{\left[ {{a_{{{\rm{K}}^ + }}}} \right]}_{{\rm{cis}}}}}},$$

where ${\left[ {{a_{{{\rm{K}}^ + }/{\rm{C}}{{\rm{l}}^ - }}}} \right]_{{\rm{cis/trans}}}}$ is the activity of the K^+^ or Cl^−^ in the *cis* or *trans* compartments, *R* the gas constant, *T* the temperature, and *F* the Faraday’s constant. We found that the ion selectivity of FraC nanopores depends on the charge of the constriction, with WtFraC being strongly cation-selective (${P_{{{\rm{K}}^ + }}}/{P_{{\rm{C}}{{\rm{l}}^ - }}}$ = 3.64 ± 0.37, pH 7.5) and ReFraC anion-selective (${P_{{{\rm{K}}^ + }}}/{P_{{\rm{C}}{{\rm{l}}^ - }}}$ = 0.57 ± 0.04, pH 7.5). Here and throughout the manuscript, errors indicate the standard deviations obtained from three or more experiments.

To gain a better understanding of the effect of pH on ion selectivity, we used a computational approach to estimate the magnitude and distribution of electrostatic field generated by nanopores when surrounded by an electrolyte at pH 7.5 and 4.5. The simulations showed that the constriction regions of WtFraC and ReFraC at the center of the nanopore exhibited highly negative and positive potentials, respectively (Fig. 2b). In WtFraC, the lowering of the pH from 7.5 to 4.5 reduced the potential at the center of the constriction by 37% (from −0.87 to −0.55 *k*_B_ *T*/*e*_*c*_, with 1 *k*_B_ *T*/*e*_*c*_ = 25.6 mV at 298 K) in line with the 43% reduction of the ion selectivity of the nanopores (${P_{{{\rm{K}}^ + }}}/{P_{{\rm{C}}{{\rm{l}}^ - }}}$ = 2.11 ± 0.23 at pH 4.5) and confirming that the ionic transport across the nanopore is dominated by the charge of the constriction. Interestingly, at pH 4.5 the ion selectivity of ReFraC increased by 37% compared to pH 7.5 (Fig. 2a) despite the roughly constant electrostatic potential of the nanopore constriction (Fig. 2b). Presumably, when the charge at the constriction does not change, the ion selectivity can still be influenced by the protonation state of other residues located on the nanopore surface (Fig. 2b).

### Electro-osmotic flow promotes polypeptides entry into FraC

The entry of the proteins inside FraC may occur by passive diffusion, and by the combined effect of the electrophoretic force on the polypeptide charges and electro-osmotic force, the latter being the results of the EOF, that is the directional flux of water across the nanopore. The strength and direction of the EOF inside a nanopore depends on its shape, charge, the nanopore asymmetry, and is related to the ion selectivity^21, 25, 26, 51–53^. An estimate of the direction and the magnitude of the water flux (*J*_*w*_) due to the ion selectivity can be obtained using the following equation^52^:

2 $${J_w} = {N_w}\frac{I}{{{e_c}}}\left( {\frac{{1 \!-\! {P_{{{\rm{K}}^ + }}}/{P_{{\rm{C}}{{\rm{l}}^ - }}}}}{{1 \!+\! {P_{{{\rm{K}}^ + }}}/{P_{{\rm{C}}{{\rm{l}}^ - }}}}}} \right),$$

where *N*_*w*_ is the number of water molecules per ion (hydration shell), *I* the ionic current, *e*_*c*_ the elementary charge, and ${P_{{{\rm{K}}^ + }}}/{P_{{\rm{C}}{{\rm{l}}^ - }}}$ the ion selectivity. Note that the equation above likely underestimates the water flux, as it does not take the movement of the electrical double layer into account. Using a value of 10 water molecules per ion^26, 52^, water fluxes and velocities can be estimated (Supplementary Table 1). In WtFraC, water flows from *cis* to *trans* at negative applied potentials and the reduced ion selectivity at pH 4.5 results in a flux reduction of ~59% (from 6.08 × 10^9^ to 2.48 × 10^9^ hydrated water molecules per second at −50 mV). The net water flux in ReFraC, on the other hand, flows from *cis* to *trans* at positive applied potentials and increases by ~51% when the pH is decreased (from 1.37 × 10^9^ at pH 7.5 to 2.08 × 10^9^ at +50 mV). These data suggest that the EOF has a dominant role in the capture of both proteins and peptides, as both chymotrypsin (25 kDa, pI = 8.8, net positive charge) and endothelin 1 (2.5 kDa, formal charge −2) enter WtFraC and ReFraC only when the direction of the EOF is from *cis* to *trans*, irrespectively from the charge of the biomarker or the sign of the applied potential at the *trans* electrode (negative for WtFraC and positive for ReFraC).

### Biomarker detection with the WtFraC nanopore

**Fig. 3:**
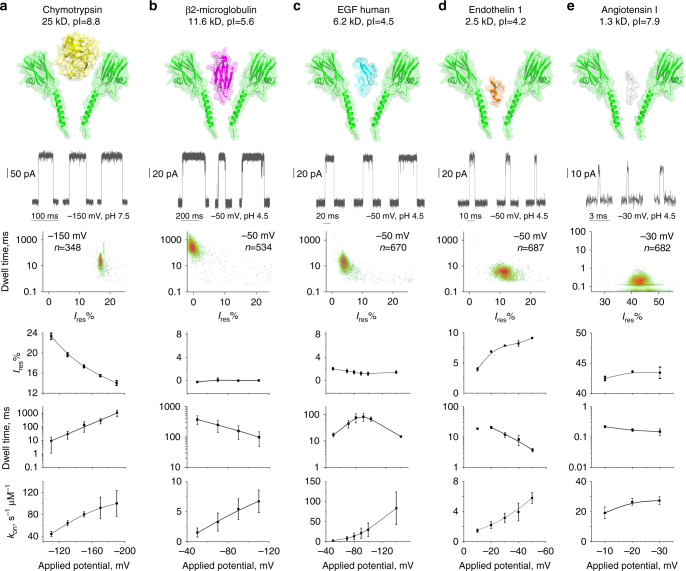
Biomarker characterization with WtFraC at pH 4.5. From top to bottom: surface representation with molecular surface and cartoon representations (PyMOL) of the biomarker, a representative trace obtained under the indicated applied potential, a heatplot depicting the dwell time distribution vs. *I*_res%_ at the same applied potential, the voltage dependence of *I*_res%_, the voltage dependence of the dwell times, and the capture frequency. **a** Chymotrypsin (25 kD, PDB: 5CHA), **b** β2-microglobulin (11.6 kD, PDB: 1LDS), **c** human EGF (6.2 kD, PDB: 1JL9), **d** endothelin 1 (2.5 kD, PDB: 1EDN), and **e** angiotensin I (1.3 kD), respectively. Angiotensin I is depicted as a random structure drawn with PyMOL. The concentrations of the biomarkers were: 200 nM for chymotrypsin, 200 nM for β2-microglobulin, 2 µM for human EGF, 1 µM for endothelin 1, and 2 µM for angiotensin I, respectively. Isoelectric points of biomarkers are obtained from literatures or with the online calculation tool PepCalc. Error bars represent the standard deviation obtained from at least 3 repeats and at least 300 events for each repeat. The voltage dependencies of capture frequencies were fitted to quadratic functions. With the exception of EGF, voltage dependencies of dwell times were fitted to single exponentials. All remaining data were fitted using a B-spline function (Origin 8.1). All recordings were collected with 50 kHz sampling rate and 10 kHz low-pass Bessel filter. Detailed numbers and analysis for each data point could be found in the supporting information (Supplementary Figs. 11–15 and Supplementary Table 7)

After assessing the capture of chymotrypsin (25 kD, 245 amino acids) and endothelin 1 (2.5 kD, 21 amino acids), biomarkers for pancreatic cysts^54^ and bronchiolitis obliterans^55^, respectively, we used the WtFraC nanopores to detect a larger range of peptide and protein biomarkers including β2-microglobulin, a 11.6 kDa (99 amino acids) biomarker for peripheral arterial disease^56^, human EGF, a 6.2 kDa (53 amino acids) biomarker for chronic kidney disease^57^, and angiotensin I, a 1.3 kD (10 amino acids) biomarker for hypertensive crisis (Fig. 3)^58^. At pH 7.5, β2-microglobulin and EGF entered the WtFraC only at high negative applied potentials (>−200 mV; Supplementary Figs. 4, 5). The entry of endothelin 1 (2.5 kD, pI = 4.16) into FraC nanopores could not be observed at potentials up to −300 mV (Supplementary Fig. 6), while the blockade of angiotensin I (1.3 kD, pI = 7.93) could not be assessed as the peptide induced blockades that were too fast to be analyzed (Supplementary Fig. 7). By contrast, at pH 4.5 all biomarkers entered the FraC nanopores. Thus all biomarkers were assessed under negative applied potentials at pH 4.5 with the exception of chymotrypsin.

### Protein translocation might deform FraC transmembrane helices

It is generally accepted^59–63^ and experimentally shown^35^ that the voltage dependence of the dwell time of a molecule can report whether it translocates a nanopore. If a molecule translocates through a nanopore, increasing the electrophoretic or electro-osmotic driving force reduces its residence time inside the nanopore (i.e., the dwell time). By contrast, if molecules do not translocate the nanopore, the dwell time will increase with the applied potential. Under this assumption, at pH 4.5 biomarkers entered and translocated WtFraC nanopores. The voltage dependence of chymotrypsin (Fig. 3a) suggests that this biomarker does not translocate through WtFraC nanopores. This is expected, giving that the protein is larger than the transmembrane constriction of the nanopore (Fig. 3a). Accordingly, the *I*_res%_, which refers to percent ratios between the blocked and open pore ionic currents, of protein blockades decreased with the applied potential, suggesting that the protein is pushed further inside the nanopore as the EOF is increased. As a folded protein β2-microglobulin is larger than the constriction of WtFraC (Fig. 3b). Surprisingly, however, we found that the dwell times of the blockades decreased with the potential, suggesting that the protein translocates through the nanopore. Translocation of β2-microglobulin would require that either the protein or the transmembrane domain of FraC unfolds or deforms. The *I*_res%_ was near zero, suggesting a tight interaction between β2-microglobulin and the nanopore walls. Further, the *I*_res%_ remained constant over the applied potential, which is consistent with a protein remaining folded within the nanopore^64, 65^. Together, these findings suggest that the transmembrane region of the nanopore deforms during the translocation of folded β2-microglobulin molecules. This interpretation is consistent with our previous study in which we observed transient remodeling of FraC transmembrane region during the translocation of double-stranded DNA through the nanopore^49^.

### Threshold potential and stretched polypeptides

The bi-modal voltage dependency of dwell times observed with EGF and endothelin 1 (Fig. 3c, d) suggests that these proteins translocate above a certain potential (−90 mV and −20 mV, respectively). This interpretation is corroborated by a previous study where the bi-modal voltage dependency of DHFR blockades to ClyA nanopores was shown to correspond to a voltage threshold potential for the translocation of the protein across the nanopore^36^. Interestingly, the *I*_res%_ of endothelin 1 increased with the applied potential, suggesting that this polypeptide may be stretched by the increased EOF through the nanopore. This observation is in accordance with previous studies reporting that proteins and polypeptides can be stretched by high applied potentials^24, 66^. If confirmed, this is an important finding for protein-sequencing applications, because it suggests that the EOF across the nanopore can linearize a polypeptide during translocation. Finally, angiotensin 1 translocated at all potentials tested (Fig. 3e). The dwell time of angiotensin 1 was close to the limit of detection, thus most likely angiotensin 1, an oligopeptide of just 10 amino acids, represents the limit of oligopeptide detection using FraC nanopores.

The capture frequency of all biomarkers increased with the applied potential. Previous work with DNA revealed that the entry of a polymer inside a nanopore can be diffusion-limited, i.e., all molecules colliding with the nanopore are captured or reaction-limited, i.e., only a fraction of molecules colliding with the nanopore are captured. For a diffusion-limited entry, polypeptide capture is expected to vary linearly with the applied voltage bias. For a reaction-limited entry, the relation should be exponential^67, 68^. Our data could not be fitted to either linear or exponential regressions (Fig. 3), suggesting that the entry and confinement of polypeptides inside FraC might be influenced by a complex interplay between the electro-osmotic, electrophoretic, and electrostatic forces inside the nanopores. By contrast, the voltage dependence of the dwell times of the polypeptide fitted well to exponential regressions (Fig. 3), indicating that the escape from the nanopore, either from the *cis* side (Chymotrypsin and EGF below −90 mV) or *trans* side (β2-microglobulin, endothelin 1, angiotensin I, and EGF above −90 mV), is a reaction-limited process.

### Recognition of peptide and protein biomarkers

**Fig. 4:**
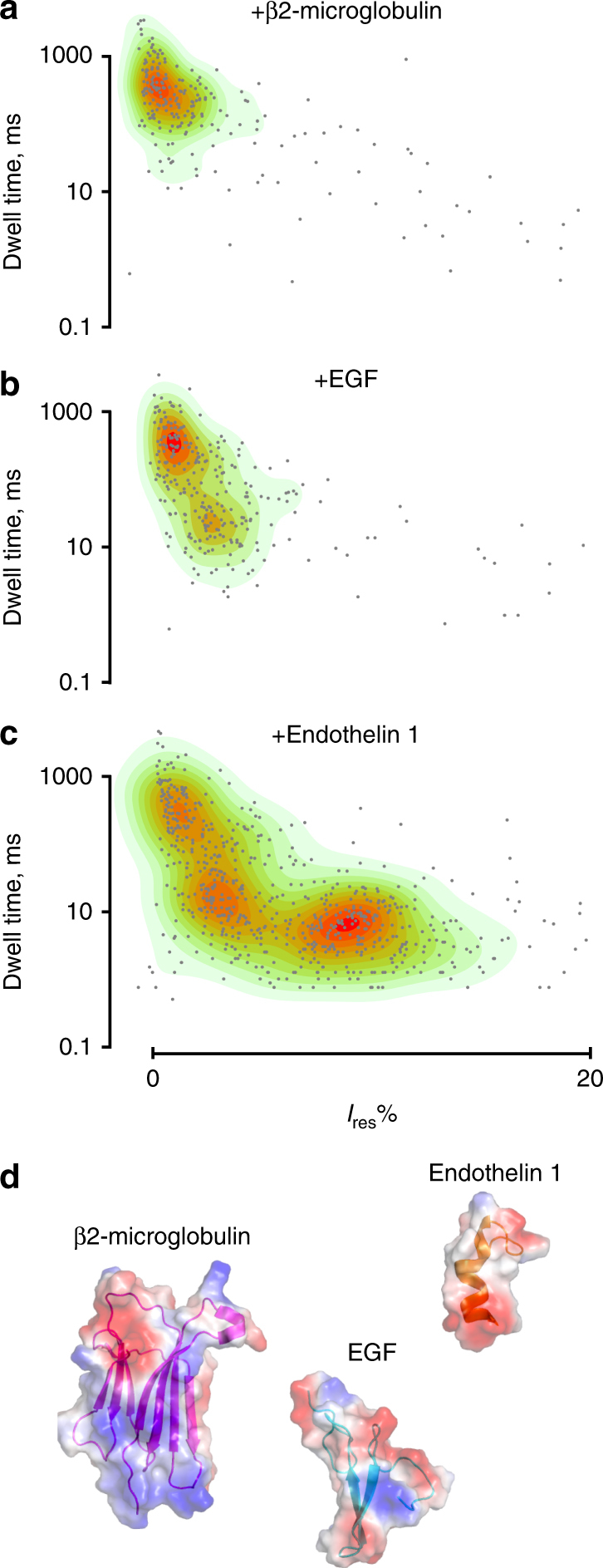
Discrimination of a biomarker mixture with WtFraC at pH 4.5. A single WtFraC nanopore was obtained in a buffer at pH 4.5 (1 M KCl, 0.1 M citric acid, 180 mM Tris base) under a −50 mV applied potential. About 200 nM of β2-microglobulin was initially added to the *cis* compartment (**a**), then 1 µM EGF (**b**), and finally 200 nM endothelin 1 (**c**) were added to *cis* compartment. **d** Crystal structure of β2-microglobulin, EGF, and endothelin 1 mixture created with PyMOL colored according to their vacuum electrostatics

**Fig. 5:**
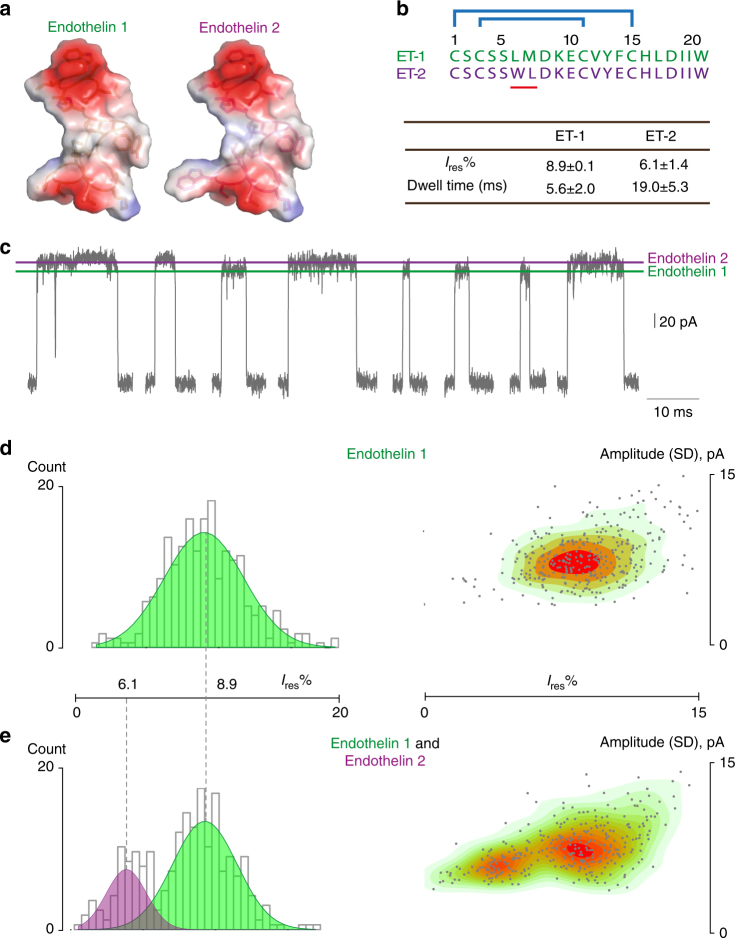
Discrimination of endothelin 1 and 2 with WtFraC at pH 4.5. **a** Molecular surface representation of endothelin 1 (PDB: 1EDN) and endothelin 2 (homology model from endothelin 1, PyMOL) using electrostatic coloring (PyMOL). **b** Above: amino-acid sequences of endothelin 1 and 2. Blue lines indicate the disulfide bridges in each polypeptide. Below: *I*_res%_ and dwell time for endothelin 1 and endothelin 2 blockades at −50 mV in pH 4.5 buffer (1 M KCl, 0.1 M citric acid, 180 mM Tris base). Standard deviations are calculated from three experiments (Supplementary Fig. 16). **c** Representative endothelin 1 and endothelin 2 blockades to the same FraC nanopore under −50 mV applied potential. **d** Histogram (left) of residual currents provoked by 2 µM endothelin 1 and corresponding heatplot depicting the standard deviation of the current amplitude vs. *I*_res%_ (right). **e** Same as in **d** but after addition of 8 µM endothelin 2 to the same pore revealing a second population. Graphs were created with custom R scripts. All recordings were conducted with 50 kHz sampling rate and 10 kHz Bessel low-pass filter

Differentially sized oligo- and polypeptides as well as proteins were easily distinguished using several parameters, including the residual current and the duration of the current blockades (Fig. 3). Using identical conditions, and the same applied voltage, we discriminated β2-microglobulin, EGF as well as endothelin 1 in a mixture (Fig. 4). At −50 mV applied potential, almost every blockade elicited by β2-microglobulin, EGF, and endothelin 1 could be distinguished. Most likely, the conical shape of FraC nanopores is instrumental for recognizing folded polymers, which presumably penetrate and interact at different heights with the lumen of the nanopore. In order to challenge our experimental system, we sought to identify even more similar analytes. We chose endothelin 1 and endothelin 2, near-isomeric polypeptides differing in 1 out of 21 amino acids being otherwise structural isomers (Fig. 5a, b, also note that leucine 6 in endothelin 1 is at position 7 in endothelin 2). Remarkably, at −50 mV, we observed distinguishable blockades with unique *I*_res%_ and dwell times (Fig. 5b, c) for endothelin 1 (*I*_res%_: 8.9 ± 0.1%, dwell time 5.6 ± 2.0 ms, *N* = 3, *n* = 600) and endothelin 2 (6.1 ± 1.4%, dwell time 19.0 ± 5.3 ms, *N* = 3, *n* = 384). This enabled their identification on an individual blockade level already (Fig. 5c). When we added consecutively first 2 µM endothelin 1 (Fig. 5d) and then 8 µM endothelin 2 to the same pore (Fig. 5e), we could also observe two distinct populations by plotting the standard deviation of the amplitude of events over their corresponding *I*_res%_. The two disulfide bonds in both endothelins likely allow them to maintain a partially folded structure also during translocation across the nanopore. Thus, the different current blockades could arise from the additional bulky tryptophan residue in endothelin 2 (Fig. 5a). By contrast, detection of smaller and unfolded oligopeptides is more challenging. Angiotensin 2, which lacks two amino acids at the C-terminal of angiotensin 1 and is expected to translocate unfolded through the nanopore, showed very short event (<100 µs at 50 kHz sampling rate). Most likely, the sequence analysis of unfolded oligopeptides will require additional improvements such as a smaller nanopore or the use of enzymes to reduce the translocation speed across the nanopore.

## Discussion

In this work, we show that FraC nanopores can be used as a sensor in single-molecule proteomic analysis. In the simplest implementation of nanopore proteomics, proteins are recognized amino acid-by-amino acid, as they translocate linearly through a nanopore. Notably, since the sequence of proteins and peptides in an organism is known from genomic analysis, a protein sequence might be obtained by recognizing only a subset of amino acids^69^. Alternatively, folded proteins and peptides could be recognized, as they reside inside the nanopore and matched to previously established blockades, i.e., matching a fingerprint-like blockade to a database of known blockades. The challenges of folded and unfolded recognition, however, are different. In folded protein recognition, the transport dynamics of analyte peptides and proteins across the nanopore are not critical, and analytes are recognized as they reside in vestibule of the nanopore. Here we showed that differentially sized oligo- and polypeptides as well as proteins were easily distinguished using several parameters including residual current and duration of the current blockade (Figs. 3, 4). Remarkably, the nanopore was also able to distinguish between blockades of endothelin 1 and endothelin 2, whose amino acid sequence only differs by a single amino acid (a tryptophan, Fig. 5). However, despite the sensitivity of nanopore currents, it is unlikely that all the proteins in a proteome will elicit a specific signal, and a biological sample will most likely require a pre-purification and/or concentration step.

An important issue in folded protein analysis is the detection of low-abundance proteins. Since the nanopore approach is a single-molecule technique, in principle the detection of low-abundance proteins is merely an issue of waiting until the target analytes are captured by the nanopore. In practice, however, the experiment should be carried out within a specific time window. Considering that 10–100 events are required to identify and quantify a specific analyte, and assuming a running time of 1000 s (about 15 min), the target analyte should be captured with a frequency of 0.1–0.01 events per second. This corresponds to a concentration threshold limit of ~1 nM for the biomarkers sampled here. Because the concentration of proteins in blood can be much lower, the sensitivity of this technique has to be increased. This could be done by using arrays of nanopores. For example an array of 1000 nanopores would allow sampling analytes in the pM range. Alternatively, binding elements such as aptamers or antibodies could be conjugated to the nanopore or to the lipid bilayer to increase the local concentration of analytes near the nanopore^23^. Finally, in analogy to most proteomic techniques, target analytes could be either purified or enriched prior analysis. Crucially, nanopore sensors only require nanoscale volumes to be sampled. Thus, the theoretical detection limit of the nanopore approach is just a few thousand copies of biomarkers collected from a biological sample.

In unfolded protein recognition, the direction of translocation of the polypeptide across the nanopore must be tightly controlled. In particular, the back movement of the polymer should be avoided, and the polypeptide should be stretched to allow addressing individual amino acids. Unlike DNA, however, polypeptides do not have a uniform charge and the electric field cannot be used to stretch or control the translocation across the nanopore. Therefore, the EOF must be used as the driving force for the nanoscale transport across a nanopore. However, since a polypeptide chain contains both positively and negatively charged amino acids, the electro-osmotic force should be higher than the electrophoretic force during translocation. An additional complication is that the EOF is generated by the fixed charge of the inner walls of the nanopore, which in turn might prevent or retard the translocation of amino acids through electrostatic interactions. In this work, we showed that at pH 7.5 none of the biomarkers we tested, most of which were negatively charged, could translocate across the WtFraC nanopore. In contrast, at pH 4.5 all the polypeptides smaller than the nanopore constriction translocated the nanopore. Since the EOF through WtFraC is reduced by ~60% upon lowering the pH to 4.5, it is likely that polypeptide translocation across the nanopore is allowed by the attenuated electrostatic potential of the constriction (reduced by ~40% in the same pH range) combined with the weaker opposing electrophoretic force on the partially protonated acidic amino acids (aspartate and glutamate) migrating toward the negative *trans* electrode. In turn, these findings suggest that at pH 4.5, the constriction of WtFraC maintains a sufficient negative charge to induce a *cis* to *trans* EOF at negative applied potentials, while allowing the translocation of the negatively charged polypeptides across the nanopore against the applied potential. Although individual amino acids could not be identified on-the-fly during translocation, we showed that differences by just one bulky tryptophan residue in a small biomarker can be observed (Fig. 5). Therefore, if the speed of transport of a polypeptide can be controlled, for example, by the use of enzymes, it might be possible that FraC nanopores will allow the identification of specific sequence features in translocating polypeptides.

## Methods

### Chemicals

α-Chymotrypsin (from bovine pancreas, ≥85%, C4129), β2-microglobulin (from human urine, ≥98%, M4890), endothelin 1 (≥97%, E7764), endothelin 2 (≥97%, E9012), angiotensin I (≥90%, A9650), pentane (≥99%, 236,705), hexadecane (99%, H6703), Trizma hydrochloride (SLBG8541V), Trizma base (SLBK4455V), N,N-Dimethyldodecylamine N-oxide (LADO, ≥99%, 40,234), and *n*-Dodecyl β-D-maltoside (DDM, ≥98%, D4641) were obtained from Sigma-Aldrich. Human EGF (≥98%, CYT-217) was obtained from PROSPEC. 1,2-diphytanoyl-sn-glycero-3-phosphocholine (DPhPC, 850,356P) and sphingomyelin (Brain, Porcine, 860,062) were purchased from Avanti Polar Lipids. Potassium chloride (≥99%, BCBL9989V) was bought from Fluka. Citric acid (≥99%, A0365028) was obtained from ACROS. All polypeptide biomarkers and chemicals were used without further purification. About 15 mM Tris base, pH 7.5 buffer used in this study was prepared by dissolving 1.902 g Trizma HCl and 0.354 g Trizma Base in 1 l water (Milli-Q, Millipore, Inc.).

### FraC monomer expression and purification

A gene containing FraC with a C-terminus 6-His tag was cloned into a pT7-SC1^70^ expression plasmid using *Nco*I and *Hin*dIII restriction digestion sites. For expression, the plasmid was transferred into *E.cloni* EXPRESS BL21(DE3) competent cell by electroporation. Transformants were harvested from the LB agar plate containing 100 mg/l ampicillin after overnight incubation at 37 °C, and inoculated into 200 ml fresh liquid 2-YT media with 100 mg/l ampicillin. The cell culture was grown at 37 °C, with 220 rpm shaking to an optical density at 600 nm of 0.8 after which 0.5 mM IPTG was added to induce expression. The temperature was lowered to 25 °C and the bacterial culture was allowed to grow for 12 h. Cells were harvested by centrifugation for 30 min (2000×*g*) at 4 °C. Cell pellets were stored at −80 °C. About 50–100 ml of cell culture pellet was thawed at room temperature, resuspended with 30 ml lysis buffer (15 mM Tris base pH 7.5, 1 mM MgCl_2_, 4 M Urea, 0.2 mg/ml lysozyme, and 0.05 units/ml DNase) and mixed vigorously with a vortex shaker for 1 h. In order to fully disrupt the cells, the suspension was sonicated for 2 min (duty cycle 10%, output control 3 using a Branson Sonifier 450). The crude lysate was then centrifuged at 5400×*g* for 20 min at 4 °C. The supernatant (containing FraC monomers) was transferred to a 50 ml falcon tube containing a 100 μl of Ni-NTA resin (Qiagen, stored at 4 °C, well mixed before pipetting out 100 μl), which was pre-washed with 3 ml of washing buffer (10 mM imidazole, 150 mM NaCl, 15 mM Tris base, pH 7.5), and incubated at room temperature for 1 h with gentle mixing. The resin was spun down at 2000×*g* for 5 min at 4 °C. Most of the supernatant was discarded and the pellet containing the Ni-NTA resin within ~5 ml of buffer was transferred to a Micro Bio-Spin column (Bio-Rad) at room temperature. The Ni-NTA beads were washed with 10 ml wash buffer and the protein was eluded with 500 μl of 300 mM imidazole. Protein concentration was determined with NanoDrop 2000 UV–Vis Spectrophotometer (Thermo Scientific). The monomers were stored at 4 °C.

### Preparation of sphingomyelin–DPhPC liposomes

About 20 mg sphingomyelin (Brain, Porcine, Avanti Polar Lipids) was mixed with 20 mg of DPhPC (Avanti Polar Lipids) and dissolved in 4 ml pentane (Sigma-Aldrich) containing 0.5% v/v ethanol. This lipid mixture was placed in a round flask and rotated slowly near a hair dryer to disperse the lipid well around the wall. The flask was kept open at room temperature for another 30 min to let the solvent evaporate completely. The lipid film deposited on the flask was then resuspended with 4 ml of buffer (150 mM NaCl, 15 mM Tris base, pH 7.5) by using a sonication bath for 5 min. The final liposome solution concentration was 10 mg/ml and stored at −20 °C.

### Oligomerization of FraC

Frozen liposomes were sonicated after thawing and mixed with monomeric FraC in a lipid:protein mass ratio 10:1. The mixture was sonicated in sonication bath ~30 s and then kept at 37 °C for 30 min. The proteo-liposome was solubilized with 0.6% LDAO (N,N-Dimethyldodecylamine N-oxide, 5% w/v stock solution in water), then transferred to a 50 ml falcon tube and diluted 20 times with buffer (150 mM NaCl, 15 mM Tris base, pH 7.5, 0.02% DDM). About 100 μl of pre-washed Ni-NTA resin (Qiagen) was added to the diluted protein/liposome mixture. After incubation with gentle shaking for 1 h, the beads were loaded to column (Micro Bio-Spin, Bio-Rad) and washed with 10 ml buffer (150 mM NaCl, 15 mM Tris base, pH 7.5). FraC oligomers were eluted with 300 µl elution buffer (200 mM EDTA, 75 mM NaCl, 7.5 mM Tris base, pH 8, 0.02% DDM). Oligomers are stable for several months at 4 °C.

### Simulation of the electrostatic potential

In order to understand the effect of pH changes on ion selectivity, we sought to simulate the magnitude and distribution of the electrostatic field created by the FraC nanopore. A well-established model for calculating such electrostatic potentials is the Poisson–Boltzmann equation (PBE):

3 $$\nabla \cdot \left[ {{\it{\epsilon }}\left( {\bf{r}} \right)\nabla \phi \left( {\bf{r}} \right)} \right] + \frac{1}{{{{\it{\epsilon }}_0}}}{\rho ^f}\left( {\bf{r}} \right) + \frac{1}{{{{\it{\epsilon }}_0}}}\mathop {\sum }\limits_{i = 1}^n {q_i}c_i^0{e^{ - \frac{{{q_i}\phi \left( {\bf{r}} \right)}}{{{k_{\rm{B}}}T}}}} = 0,$$

where $\phi \left( {\bf{r}} \right)$ is the electrostatic potential, ${\it{\epsilon }}\left( {\bf{r}} \right)$ the relative permittivity, and ${\rho ^f}\left( {\bf{r}} \right)$ the distribution of fixed atomic charges, which are all dependent on positional vector **r**. The symbols *ϵ*_0_, *k*_B_, and *T* represent the permittivity of free space, the Boltzmann constant, and the temperature in kelvins, respectively. Each mobile ion species *i* of the electrolyte is represented by their net charge *q*_*i*_ and their bulk concentration $c_i^0$.

When calculating the fixed charge distribution *ρ* ^*f*^ of the nanopores, the individual p*K*a values of all titratable groups (ASP, GLU, TYR, HIS, ARG, LYS, and the C- and N-termini) were estimated with PROPKA^71^ (Supplementary Tables 2, 3). A modified version of the PDB2PQR^72^ software was then used to assign a radius and pH-dependent partial charge to each atom in the model (“PQR” file format), taking into account the partial (de)protonated states of any residue whose p*K*a was close to the given pH. First, the average protonated fraction (*f*_HA_) of a residue at a given pH was calculated: ${f_{{\rm{HA}}}} = {\left( {1 + {{10}^{{\rm{pH}} - {\rm{p}}K{\rm{a}}}}} \right)^{ - 1}}$. Next, the partial charge of each atom in the residue (*δ*) was adjusted proportionally to the average protonation state: $\delta = {\delta _{{\rm{HA}}}} \times {f_{{\rm{HA}}}} + {\delta _{{{\rm{A}}^ - }}} \times \left( {1 - {f_{{\rm{HA}}}}} \right)$. Here *δ*_HA_ and ${\delta _{{{\rm{A}}^ - }}}$ represent the partial charge of the atom in the protonated and the deprotonated states of the amino acid, respectively. Atomic charges and radii were based on the PARSE force field.

The homology models WtFraC and ReFraC were built from the FraC crystal structure (4TSY)^50^ using the VMD^73^ and MODELLER^74^ software packages.

The Adaptive Poisson–Boltzmann Solver (APBS)^75^ was then used to calculate the electrostatic potential maps for WtFraC and ReFraC at pH 4.5 and 7.5 in 1 M KCl. Briefly, each PQR file was processed by APBS and the “draw_membrane2” program (included with APBS) to set up and solve the full PBE in three sequential calculations with increasing precision (Supplementary Fig. 8). The nanopore molecular surface (1.4 Å probe) was used as the barrier between protein interior (*ϵ* = 10, ion-inaccessible) and the electrolyte (*ϵ* = 80). A lipid bilayer was included in the form of a 3 nm-thick, dielectric slab (*ϵ* = 2, ion-inaccessible) located at the center of FraC’s transmembrane domain as determined in the OPM database^76^. The monovalent salt concentration was set to 1 M and the radius of both ions was 2.0 Å. First, a coarse calculation was performed with a large box to mitigate boundary effects and to ensure proper conversion (600 × 600 × 600 Å^3^ size, 1.86 Å resolution, ${\varphi _{{\rm{edge}}}} = 0$). The coarse solution was then used in two sequential “focussing” calculations with a medium (300 × 300 × 300 Å^3^ size, 0.93 Å resolution, ${\varphi _{{\rm{edge}}}} = {\varphi _{{\rm{coarse}}}}$) and a fine box (150 × 150 × 150 Å^3^ size, 0.47 Å resolution, ${\varphi _{{\rm{edge}}}} = {\varphi _{{\rm{medium}}}}$). Further refinement of the grid did not result in a quantitatively different result. A sub-unit averaged electrostatic map was obtained by performing the same calculation eight times while rotating the atomic coordinates of the pore in steps of 45° around the *z*-axis between each calculation and subsequently averaging the resulting electrostatic maps. Cross-sectional slices were plotted using PyMOL.

### Electrical recording in planar lipid bilayers

Two compartments of the electrophysiology chamber were separated by a 25 µm polytetrafluoroethylene film (Goodfellow Cambridge Limited) containing an aperture with a diameter of about 100 μm. To form a lipid bilayer, ~5 μl of hexadecane solution (10% v/v hexadecane in pentane) was added to the polytetrafluoroethylene film. After ~2 min, 0.5 ml buffer was added to each compartment and 10 μl of a 10 mg/ml solution of DPhPC, dissolved in pentane, directly added on top of the solutions. After brief waiting to allow for evaporation of pentane, silver/silver-chloride electrodes were submerged into each compartment. The ground electrode was connected to the *cis* compartment, the working electrode to *trans* side. A lipid bilayer spontaneously forms by lowering the buffer above and below the aperture in the polytetrafluoroethylene film. FraC oligomers were added to the *cis* side. Under an applied potential, the ionic current of FraC is asymmetric, allowing the determination of the orientation of FraC nanopores in the lipid bilayer. WtFraC nanopores showed the orientation as shown in Fig. 1, Supplementary Fig. 9, and Supplementary Table 4 when a higher conductance was measured at negative applied potential. Analytes were then added to *cis* compartment. Two kinds of buffer solutions were used for electrophysiology recording in this study depending on the pH. At pH 7.5, recordings were performed using 1 M KCl and 15 mM Tris base. When the pH was varied from 7.5 to 4.5, the buffer used contained 1 M KCl, 0.1 M citric acid, and 180 mM Tris base. FraC and ReFraC oligomers could insert into lipid bilayer from pH 4.5 to 7.5.

### Data recording and analysis

Planar bilayer recordings were collected using a patch clamp amplifier (Axopatch 200B, Axon Instruments) and the data digitized with a Digidata 1440 A/D converter (Axon Instruments). Data were acquired by using Clampex 10.4 software (Molecular Devices) and the subsequent analysis was carried out with Clampfit software (Molecular Devices). Events duration (dwell time), time between two events (inter-event time), blocked current levels (*I*_B_), and open pore levels (*I*_O_) were detected by “single channel search” function. *I*_res_%, defined as (*I*_B_/*I*_O_) × 100, was used to describe the extent of blockade caused by different biomarkers. Average inter-event times were calculated by binning the inter-event times and applying a single exponential fit to cumulative distributions.

### Ion selectivity measurement

The ion permeability ratio (K^+^/Cl^−^) was calculated using the Goldman−Hodgkin−Katz equation (Eq. 1), which uses the reverse potential (*V*_r_) as variable input. The activity of KCl at 1960 and 467 mM was calculated using mean activity coefficients for 2000 and 500 mM KCl, respectively^77^. The *V*_r_ was measured from extrapolation from I–V curves collected under asymmetric salt concentration condition. Individual FraC nanopores were reconstituted using the same buffer in both chambers (symmetric conditions, 840 mM KCl, 15 mM Tris base, pH 7.5, 500 µl) to assess the orientation of the nanopore. About 400 µl solution containing 3.36 M KCl, 15 mM Tris base, pH 7.5 was slowly added to *cis* chamber and 400 µl of a buffered solution containing no KCl (15 mM Tris base, pH 7.5) was added to *trans* solution (*trans*:*cis*, 467 mM KCl: 1960 mM KCl). The solutions were mixed and I–V curves collected from −30 to 30 mV with 1 mV steps. Experiments at pH 4.5 were carried out using the same method but using 0.1 M citric acid buffered solutions. Initially, 500 µl 840 mM KCl, 0.1 M citric acid, 180 mM Tris base buffer was added into both chambers and a single FraC channel obtained. Then, 400 µl of pH 4.5 solution containing 3.36 M KCl, 0.1 M citric acid, 180 mM Tris base was slowly added to *cis* chamber and 400 µl of a buffered solution containing no KCl (0.1 M citric acid, 180 mM Tris base, pH 4.5) was added to *trans* solution (thus yielding a *trans*:*cis* ratio of 467 mM KCl: 1960 mM KCl). The solutions were mixed and I–V curves collected from −30 to 30 mV with 1 mV steps. The directionality of the ion selectivity was also tested by using high KCl concentration in *trans* chamber and low KCl concentration in the *cis* chamber (Supplementary Fig. 10; Supplementary Tables 5, 6). Ag/AgCl electrodes were surrounded with 2.5% agarose bridges containing 2.5 M NaCl.

### Data availability

The authors declare that the data supporting the findings of this study are available within the article and its Supplementary Information files or from the corresponding authors upon reasonable request.

## Electronic supplementary material

Supplementary Information

Peer Review File
