# Supplementary material for: Electro-osmotic capture and ionic discrimination of peptide and protein biomarkers with FraC nanopores
Source: Nat Commun. 2017 Oct 16;8:935. doi: 10.1038/s41467-017-01006-4 (PMC5715100; doi:10.1038/s41467-017-01006-4)
Supplement: Supplementary file 1 — Supplementary Information [file 41467_2017_1006_MOESM1_ESM.pdf]

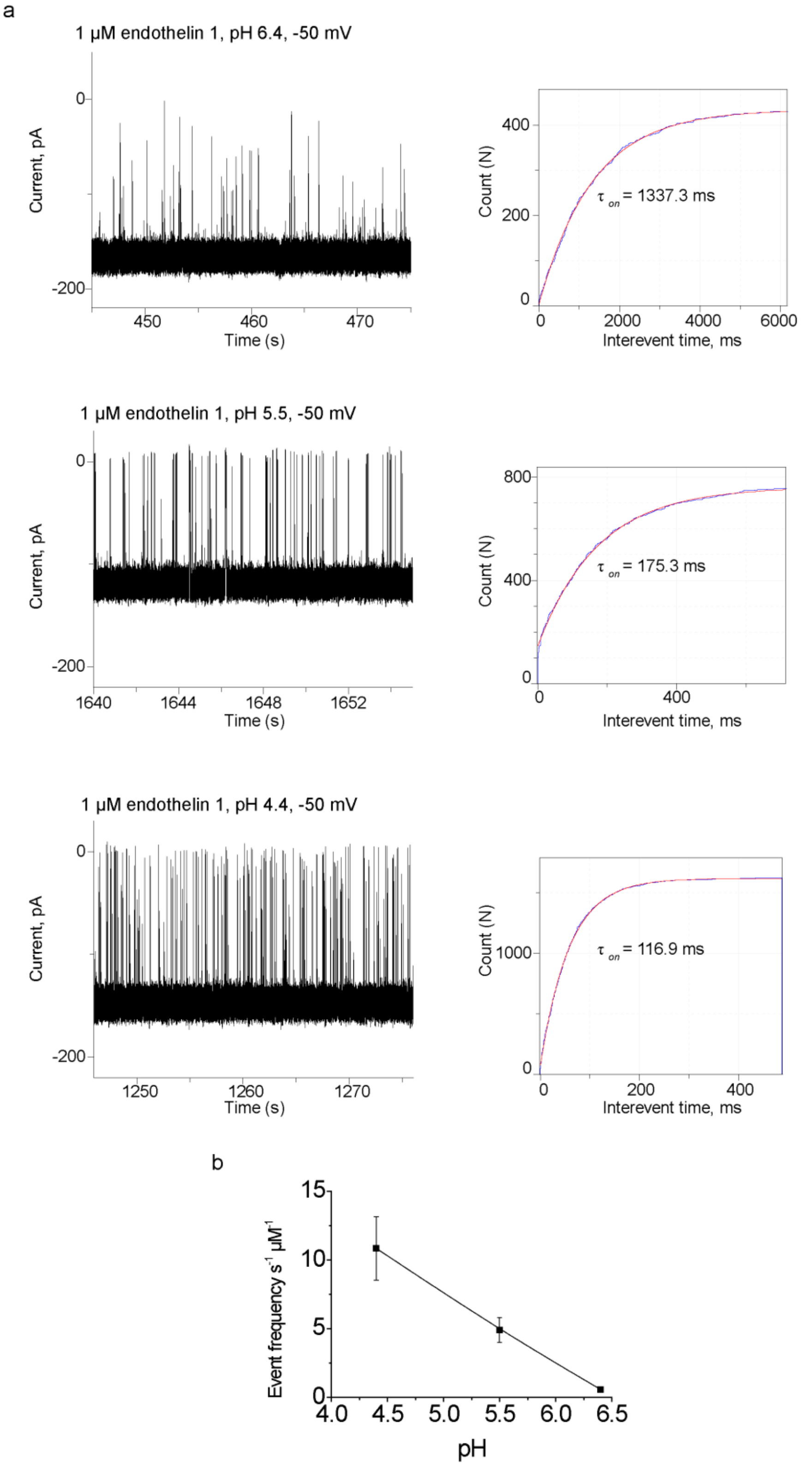

Supplementary Figure 1. pH dependency of endothelin 1 capture frequency by WtFraC under -50 mV applied potentials. (**a**) 1 µM endothelin 1 was added into the *cis*

compartment. The initial buffer contained 1 M KCl, 0.1 M citric acid, 100 mM Tris base and was titrated with 2 M NaOH to pH 7.5. Sequential addition of 1.5 µl, 3.5 µl, 7 µl of 6 M of HCl to both compartments (containing 500 µl starting buffer) decreased the pH to 6.4, 5.5 and 4.4 respectively. Events were collected with a 50 kHz sampling rate and 10 kHz low-pass Bessel filter. To the right of the current traces are shown the single exponential fittings (red lines) to cumulative distributions (blue lines) of the collected event inter-event times. (**b**) The dependence of the inter-event times with the pH. Errors are standard deviations from 3 independent repeats.

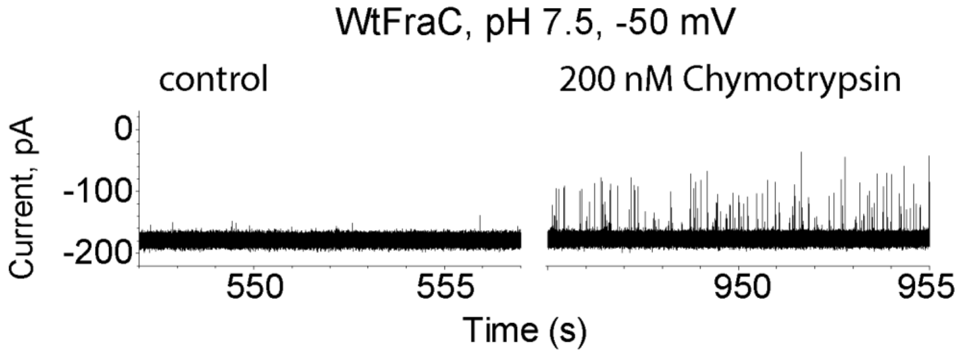

Supplementary Figure 2. Blockades elicited by 200 nM chymotrypsin at -50 mV. Chymotrypsin was added into the *cis* compartment and the buffer (1 M KCl, 15 mM Tris base, pH 7.5) was used. Recordings were obtained using 50 kHz sampling rate and a 10 kHz low-pass Bessel filter.

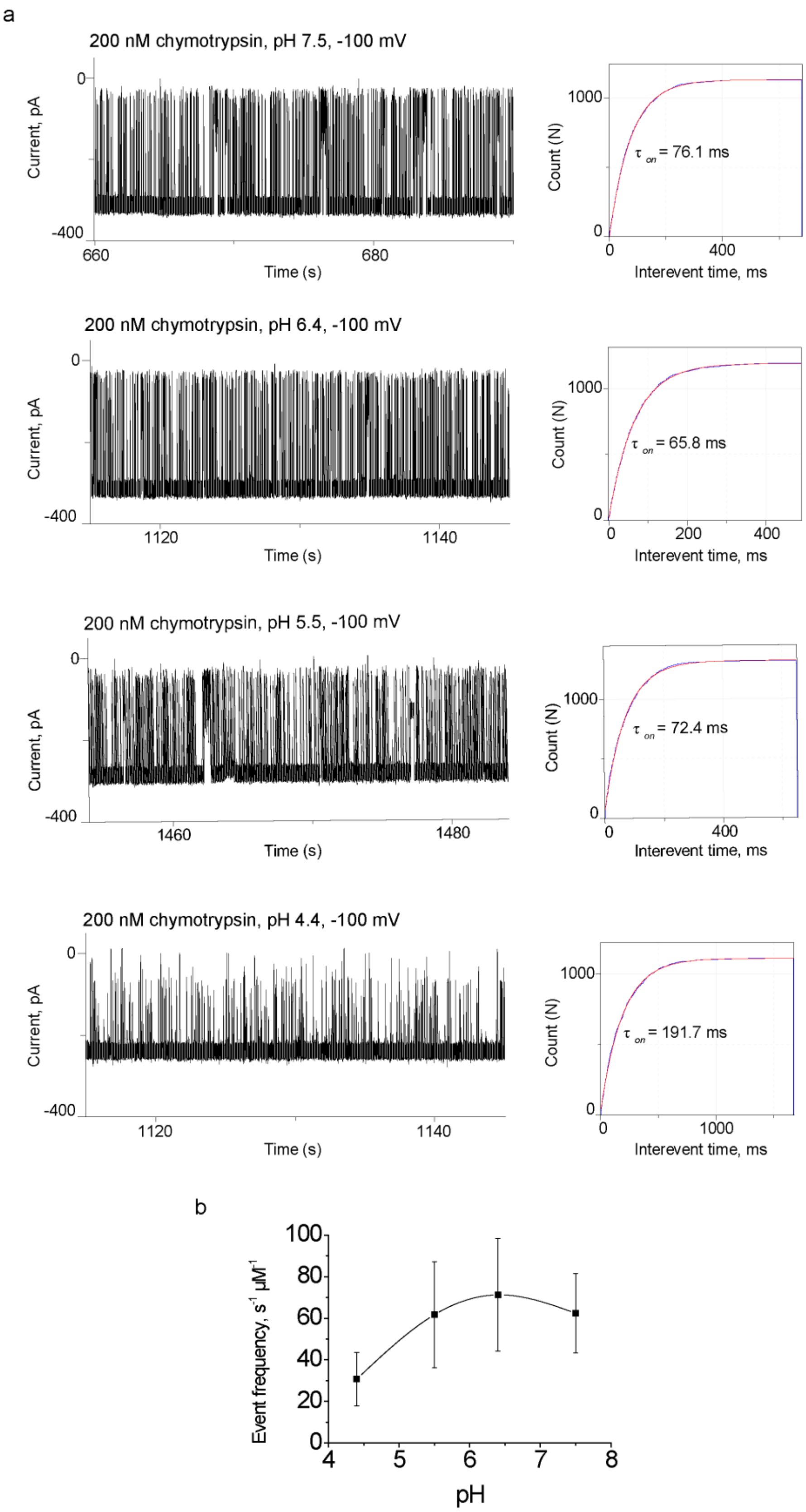
a
200 nM chymotrypsin, pH 7.5, -100 mV
Current, pA
Time (s)
Count (N)
τ on = 76.1 ms
Interevent time, ms
200 nM chymotrypsin, pH 6.4, -100 mV
τ on = 65.8 ms
200 nM chymotrypsin, pH 5.5, -100 mV
τ on = 72.4 ms
200 nM chymotrypsin, pH 4.4, -100 mV
τ on = 191.7 ms
b
Event frequency, s-1 μM-1
pH

Supplementary Figure 3. pH dependency of chymotrypsin capture by WtFraC under -100 mV applied potential. (**a**) 200 nM chymotrypsin was added to the *cis* compartment of a WtFraC nanopore. The initial buffer was 1 M KCl, 0.1 M citric acid, 100 mM Tris base at pH 7.5. Sequential addition of 1.5 µl, 3.5 µl, 7 µl of 6 M HCl to both compartments (containing 500 µl starting buffer) decreased the pH to 6.4, 5.5 and 4.4 respectively. Events were collected with a 50 kHz sampling rate and 10 kHz low-pass Bessel filter. To the right of the current traces are shown the single exponential fittings (red lines) to cumulative distributions (blue lines) of the collected event inter-event times. (**b**) The dependence of the inter-event times with the pH. Errors are standard deviations from 3 repeats.

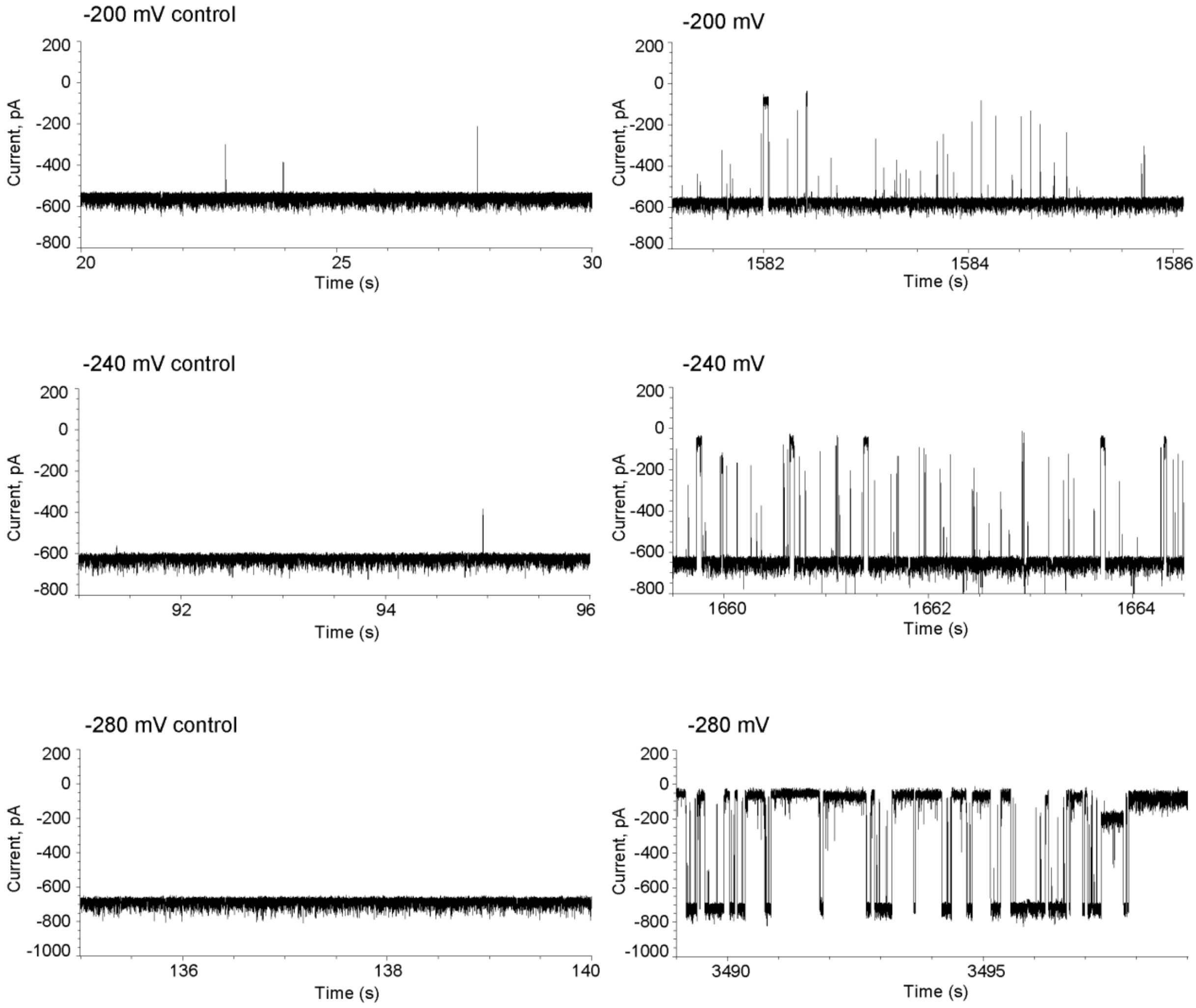

Supplementary Figure 4. β2-microglobulin blockades to WtFraC at pH 7.5 under increasing bias. The buffer was 1 M KCl, 15 mM Tris base, pH 7.5 and β2-microglobulin (200 nM) was added into *cis* side. Events were recorded with a 10 kHz sampling rate and 2 kHz low-pass Bessel filter.

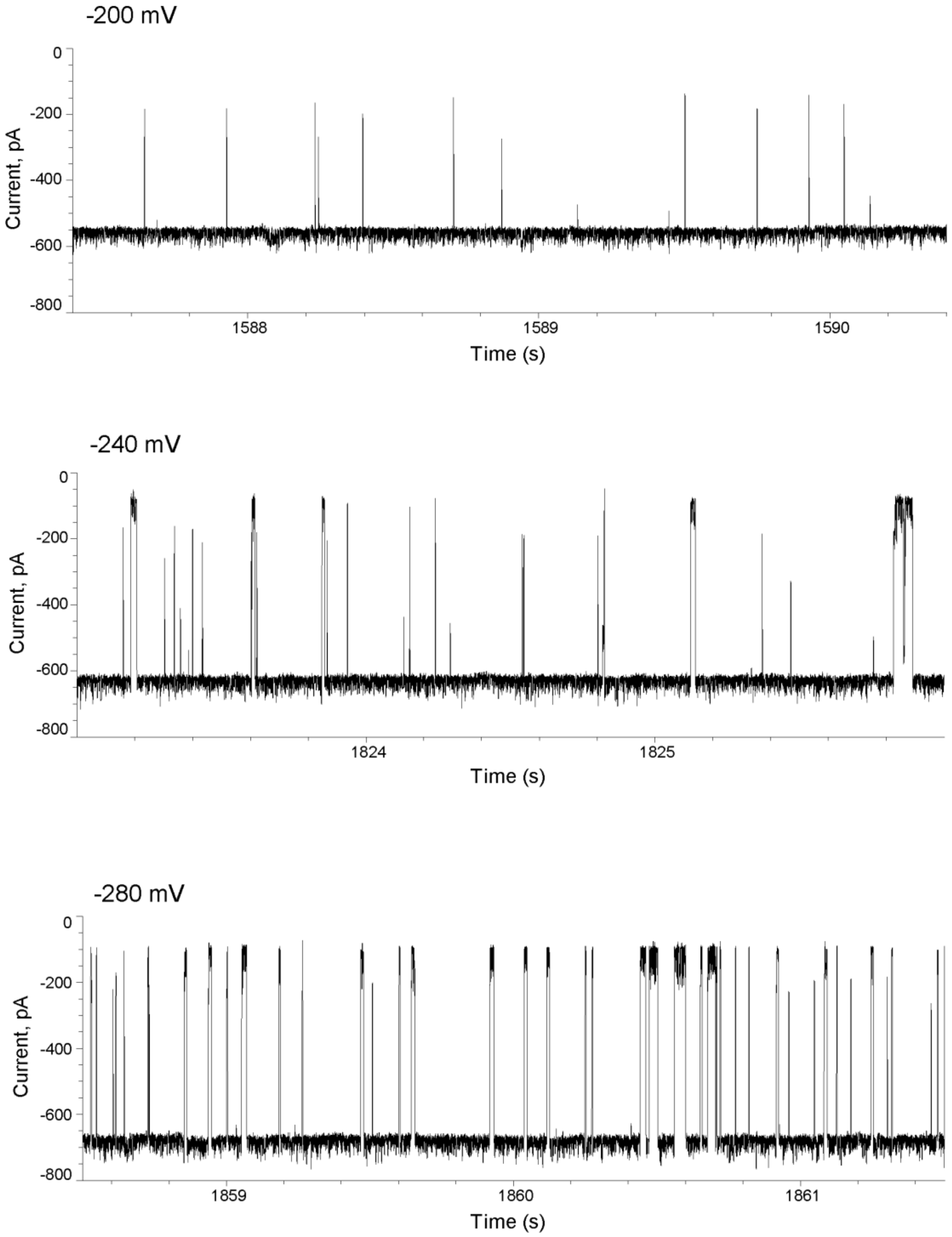

Supplementary Figure 5. EGF blockades to WtFraC at pH 7.5 under increasing bias. The buffer was 1 M KCl, 15 mM Tris base, pH 7.5. EGF (200 nM) was to the *cis* side. The time scale for the traces is the same for all traces (3 s). Events were recorded with a 10 kHz sampling rate and 2 kHz low-pass Bessel filter.

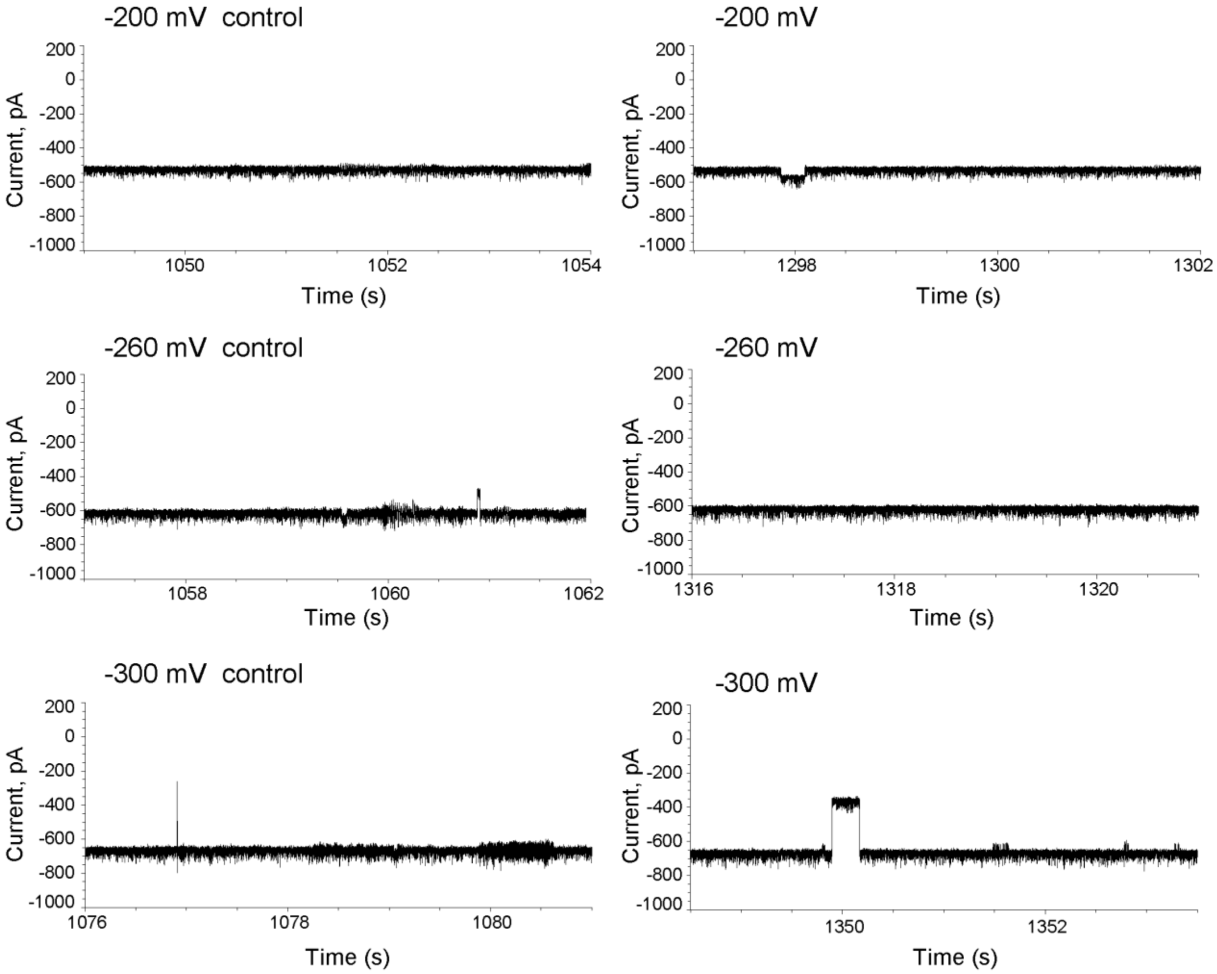

Supplementary Figure 6. Endothelin 1 blockades to WtFraC at pH 7.5 under increasing potentials. The buffer was 1 M KCl, 15 mM Tris base, pH 7.5. Endothelin 1 (200 nM) was added to the *cis* of ClyA. The time scale for all traces is 5 s. Events were recorded with a 10 kHz sampling rate and 2 kHz low-pass Bessel filter.

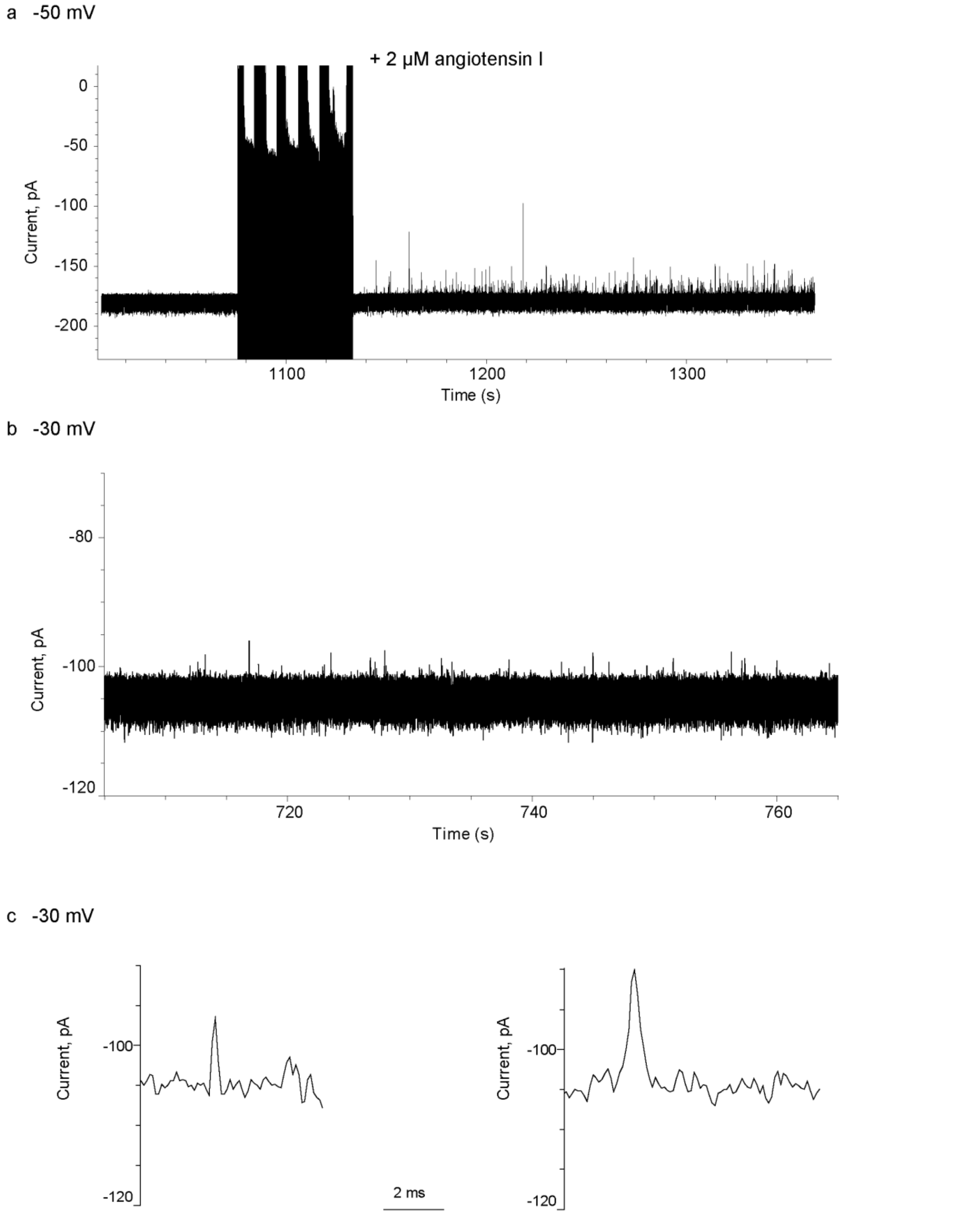

Supplementary Figure 7. Angiotensin I blockades to WtFraC at pH 7.5. The buffer was 1 M KCl, 15 mM Tris base, pH 7.5. Angiotensin I (2 µM) was added to *cis*. (**a**) Trace under -50 mV before and after addition of angiotensin I; (**b**) Trace at -30 mV; (**c**) Zoom in to show the blockade trace at -30 mV. Events were recorded with a 50 kHz sampling rate and 10 kHz low-pass Bessel filter.

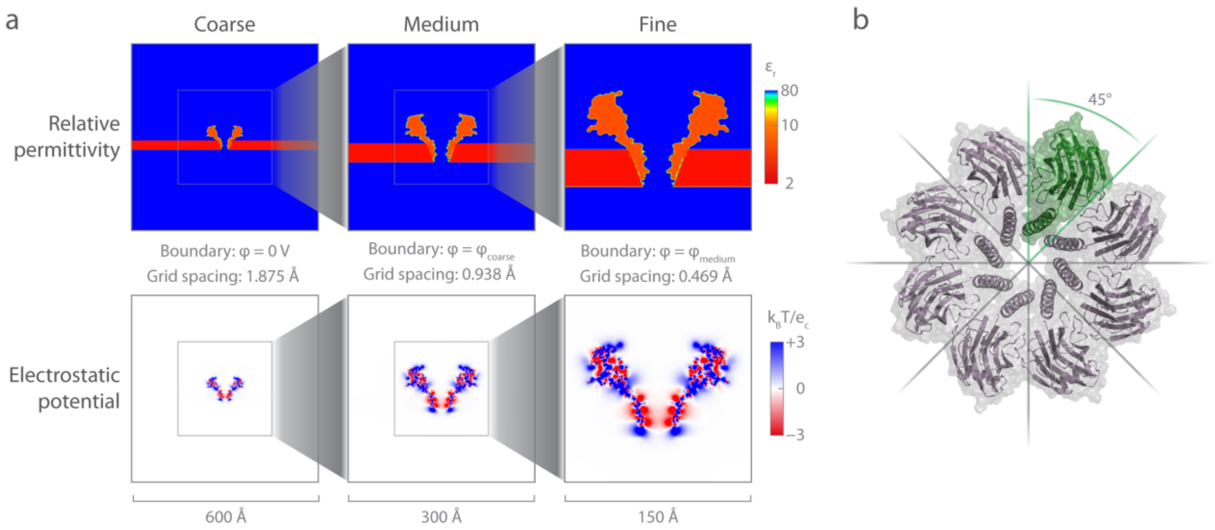

Supplementary Figure 8. Using sequential focussing to refine the electrostatic potential calculation of the 8-fold rotationally symmetric FraC nanopore. (**a**) Cross-sections through the central XZ-plane of the 3D grids of the relative permittivity (top) and electrostatic potential (bottom) for the coarse, medium and fine Poisson-Boltzmann calculations. By using a larger but less accurate solution as boundary condition for a finer calculation, it is possible to produce accurate results at a low computational cost. (**b**) Highlighted top-down view of a single sub-unit of the FraC nanopore, showing its 8-fold rotationally symmetry. Slices and cartoons were rendered with PyMOL.

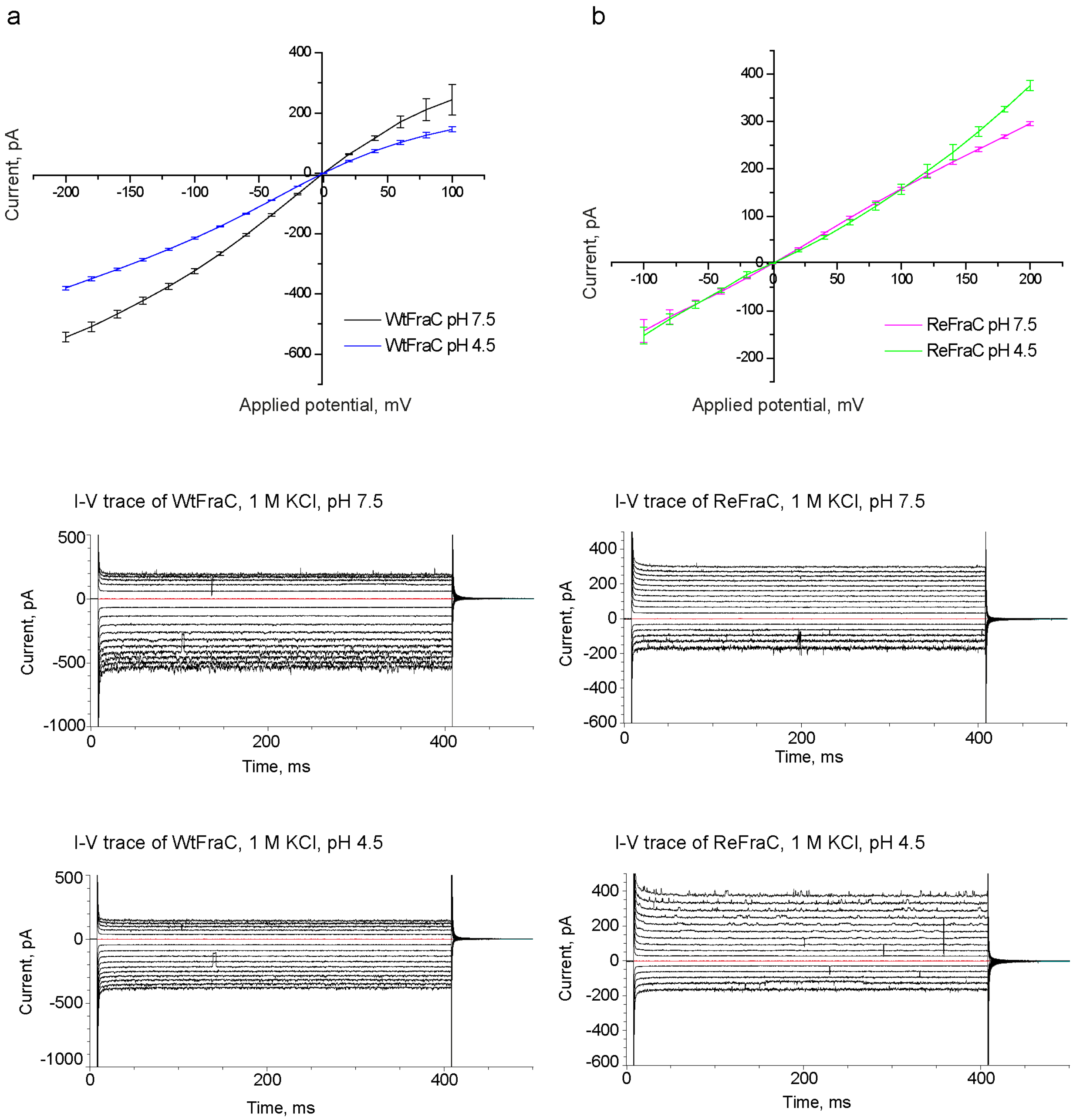

Supplementary Figure 9. Current-voltage (I-V) curves of WtFraC and ReFraC at pH 7.5 and pH 4.5. (**a**) Above: WtFraC at pH 7.5 (black), and pH 4.5 (blue). Below: Examples of a current trace output from an automated protocol where the potential is changed from +100 mV to -200 mV in 20 mV steps. (**b**) Same as in (**a**) but for ReFraC at pH 7.5 (pink), and pH 4.5 (green). The pH 7.5 buffers contained 1 M KCl buffer and 15 mM Tris base and or pH 4.5 buffer refers to 1 M KCl, 0.1 M citric acid, 180 mM Tris base. The values of I-V values are shown in Supplementary Table 7. Error bars represent the standard deviation from 3 repeats.

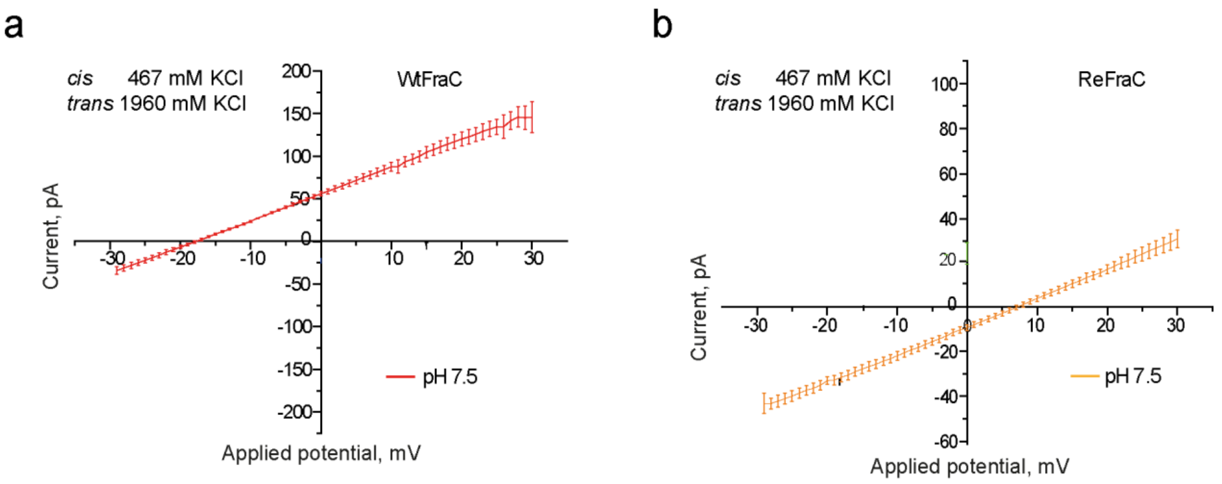

c

	WtFraC		ReFraC	
	V_r, mV	P_{K^+} / P_{Cl^-}	V_r, mV	P_{K^+} / P_{Cl^-}
pH 7.5	-17.9±0.58	3.85±0.21	7.5±0.88	0.60±0.04

Supplementary Figure 10. Ion selectivity of WtFraC and ReFraC with high ionic strengths on the *trans* side at pH 7.5. (**a, b**) Current-voltage (*IV*) curves of WtFraC nanopores (**a**) and ReFraC nanopores (**b**). The buffer contained 15 mM Tris and the pH set to 7.5. The *trans* solution contained 1960 mM KCl and the *cis* solution contained 467 mM KCl. (**c**) Values of reversal potentials (V_r) and calculated ion-selectivity (P_{K^+}/P_{Cl^-}) according to the Goldman-Hodgkin-Katz equation (equation 1 in the main text). Electrophysiology recordings were carried out using 10 kHz sampling rate and 2 kHz low-pass Bessel filter. Errors are given as standard deviations calculated from 3 experiments.

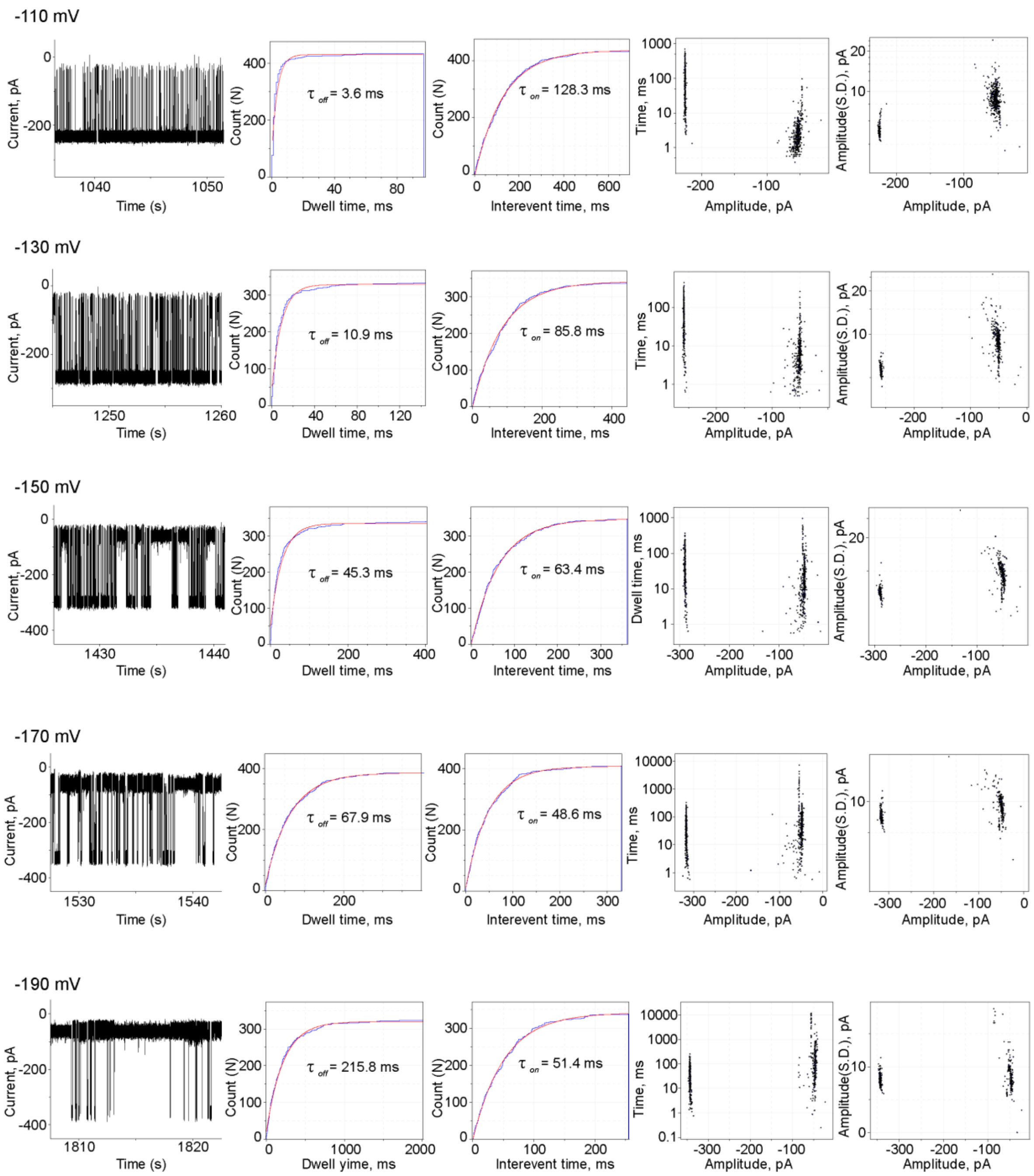

Supplementary Figure 11. Voltage dependence of chymotrypsin (200 nM) blockades to WtFraC at pH 7.5. From left to right: 15 seconds of a typical current trace, single exponential fits (red lines) to cumulative distributions (blue lines) of the dwell times and inter-event times, dwell-time versus the amplitude of the blockades, and standard deviation (S.D.) of the amplitude versus the amplitude of the blockades. The buffer was 1 M KCl, 15 mM Tris base (pH 7.5). Events were recorded with a 50 kHz sampling rate and 10 kHz low-pass Bessel filter. The amplitude standard deviation (S.D.) was given by Clampfit during the single-channel search (Molecular devices).

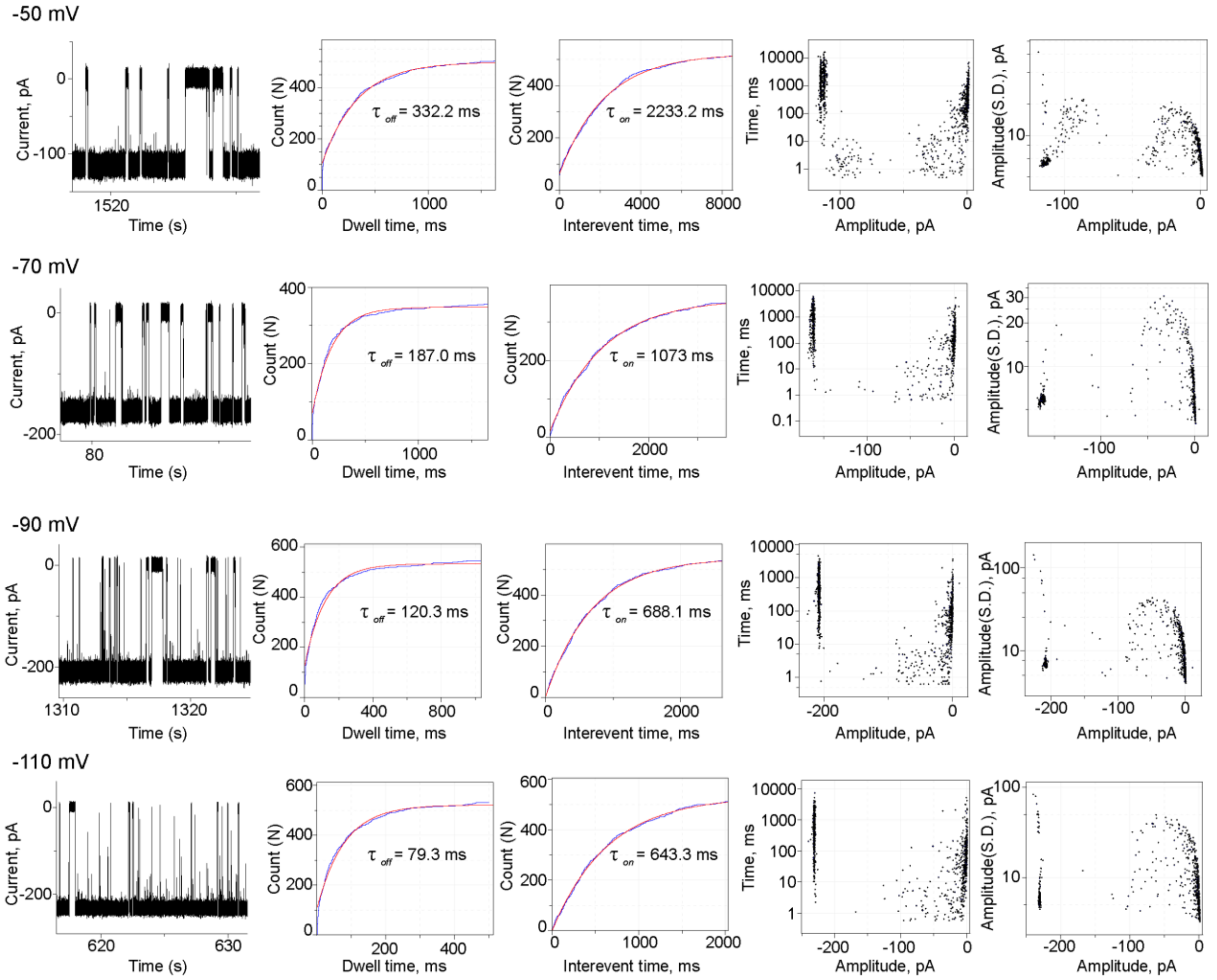

Supplementary Figure 12. Voltage dependence of β2-microglobulin (200 nM) induced blockades to WtFraC at pH 4.5. From left to right: 15 seconds of a typical current trace, single exponential fits (red lines) to cumulative distributions (blue lines) of the dwell times and inter-event times, dwell-time versus the amplitude of the blockades, and amplitude standard deviation (S.D.) the versus the amplitude of the blockades. The buffer was 1 M KCl, 0.1 M citric acid, 180 mM Tris Base (pH 4.5). Events were recorded with a 50 kHz sampling rate and 10 kHz low-pass Bessel filter. Amplitude standard deviations were given by the Clampfit software (Molecular devices).

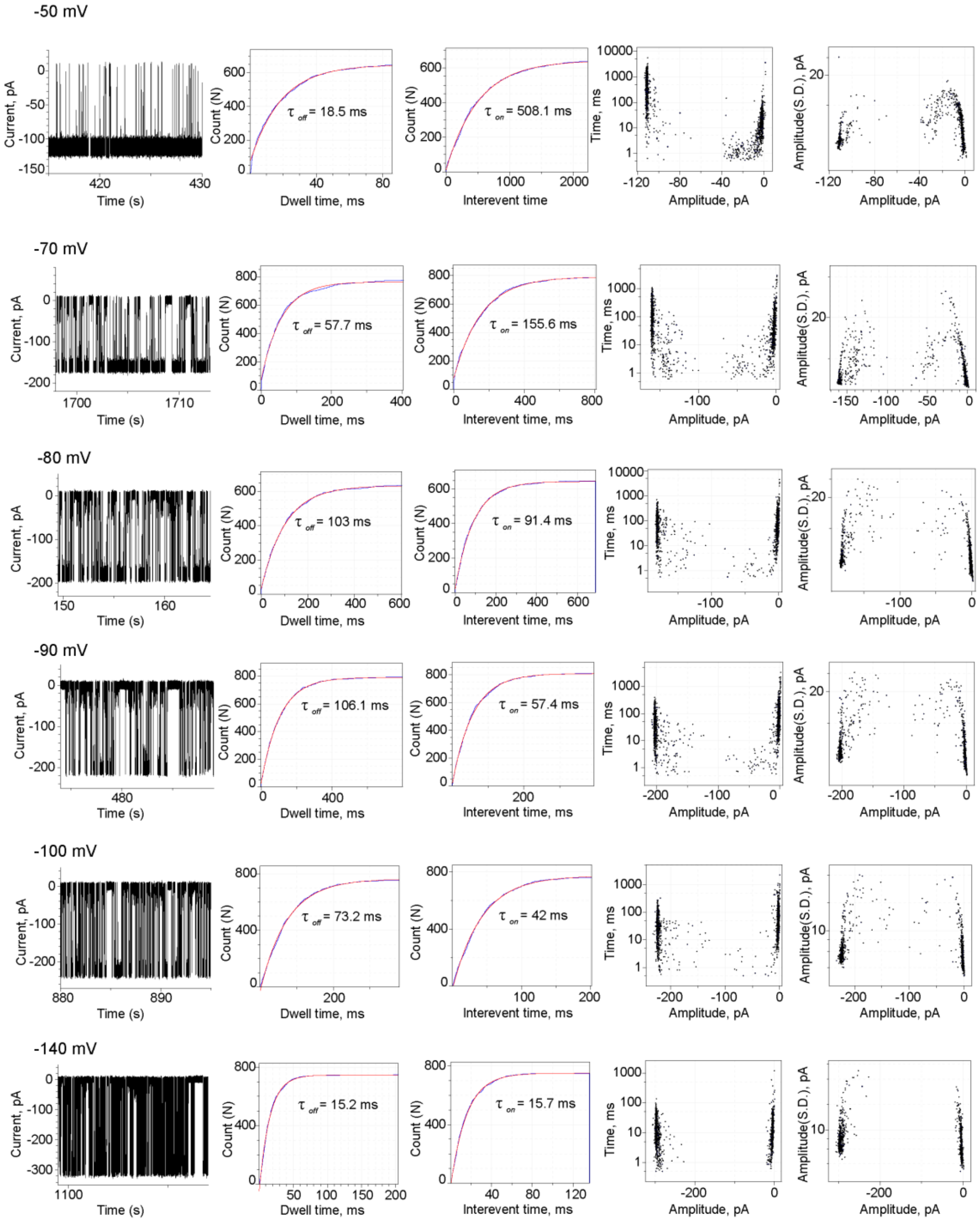

Supplementary Figure 13. Voltage dependence of human EGF (1 μM) induced blockades to WtFraC at pH 4.5. From left to right: 15 seconds of a typical current trace, single exponential fits (red lines) to cumulative distributions (blue lines) of the dwell times and inter-event times, dwell-time versus the amplitude of the blockades, and standard deviation (S.D.) of the amplitude versus the amplitude of the blockades. The buffer was 1 M KCl, 0.1 M citric acid, 180 mM Tris base (pH 4.5). Events were recorded with a 50 kHz sampling rate and 10 kHz low-pass Bessel filter. Amplitude standard deviation (S.D.) was given by the Clampfit during the single-channel search (Molecular devices).

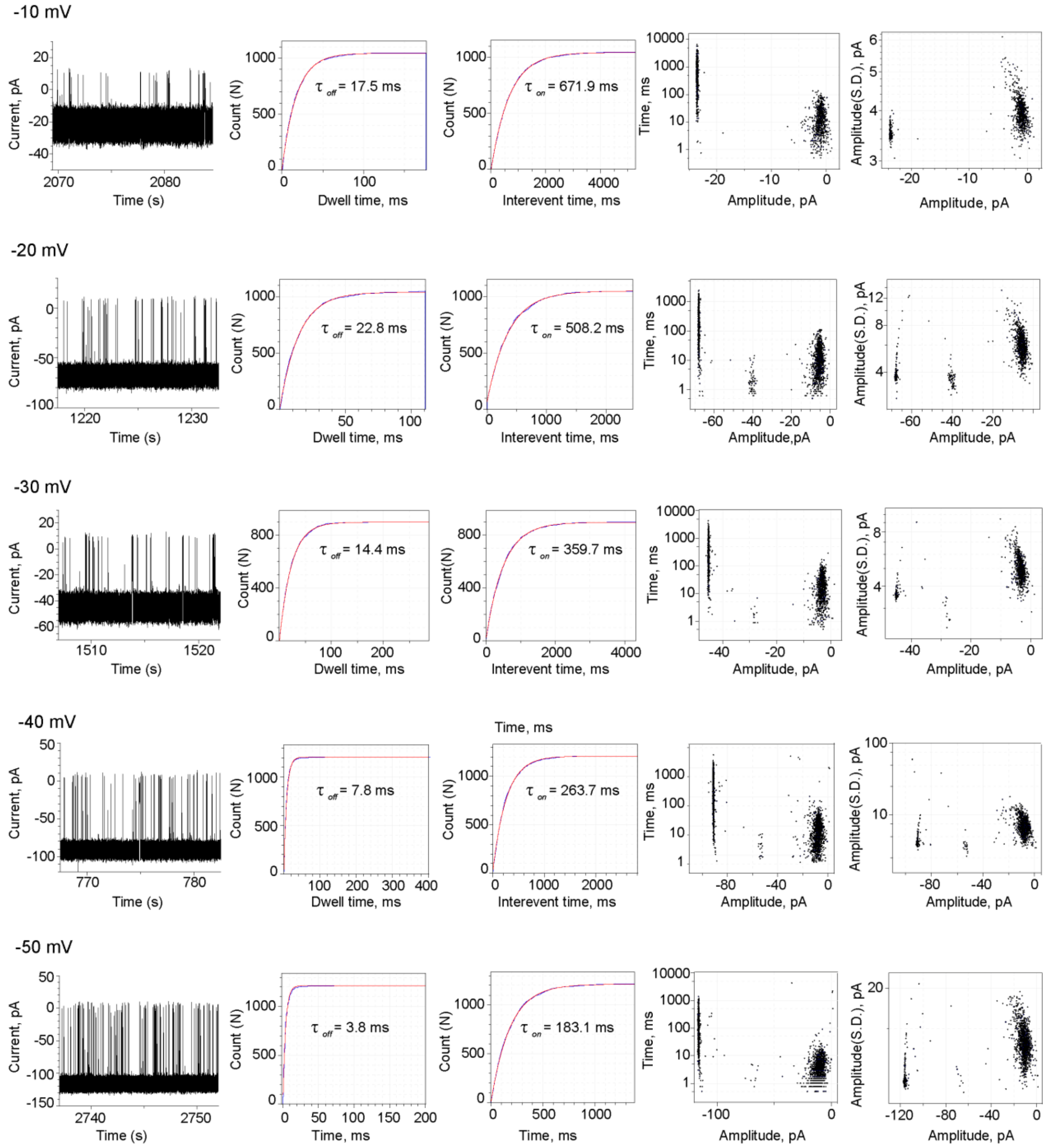

Supplementary Figure 14. Voltage dependence of endothelin 1 (200 nM) induced blockades to WtFraC at pH 4.5. From left to right: 15 seconds of a typical current trace, single exponential fits (red lines) to cumulative distributions (blue lines) of the dwell times and inter-event times, dwell-time versus the amplitude of the blockades, and amplitude standard deviation (S.D.) the versus the amplitude of the blockades. The buffer was 1 M KCl, 0.1 M citric acid, 180 mM Tris base (pH 4.5). Events were recorded with a 50 kHz sampling rate and 10 kHz low-pass Bessel filter. Amplitude standard deviations (S.D.) were given by the Clampfit during the single-channel search (Molecular devices).

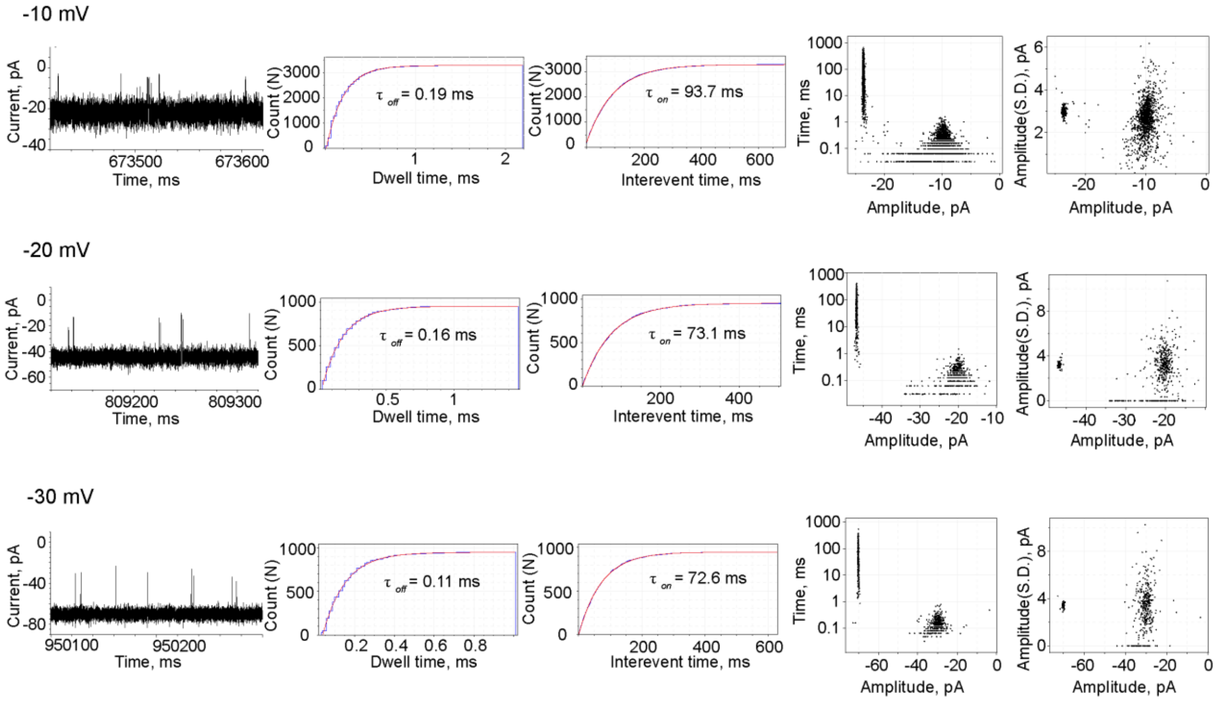

Supplementary Figure 15. Voltage dependence of angiotensin I (2 µM) blockades to WtFraC at pH 4.5. From left to right: 15 seconds of a typical current trace, single exponential fits (red lines) to cumulative distributions (blue lines) of the dwell times and inter-event times, dwell-time versus the amplitude of the blockades, and amplitude standard deviation (S.D.) of the amplitude versus of the blockades. The buffer was 1 M KCl, 0.1 M citric acid, 180 mM Tris base (pH 4.5). Events were recorded with a 50 kHz sampling rate and 10 kHz low-pass Bessel filter. Amplitude standard deviations (S.D.) were given by the Clampfit during the single-channel search (Molecular devices).

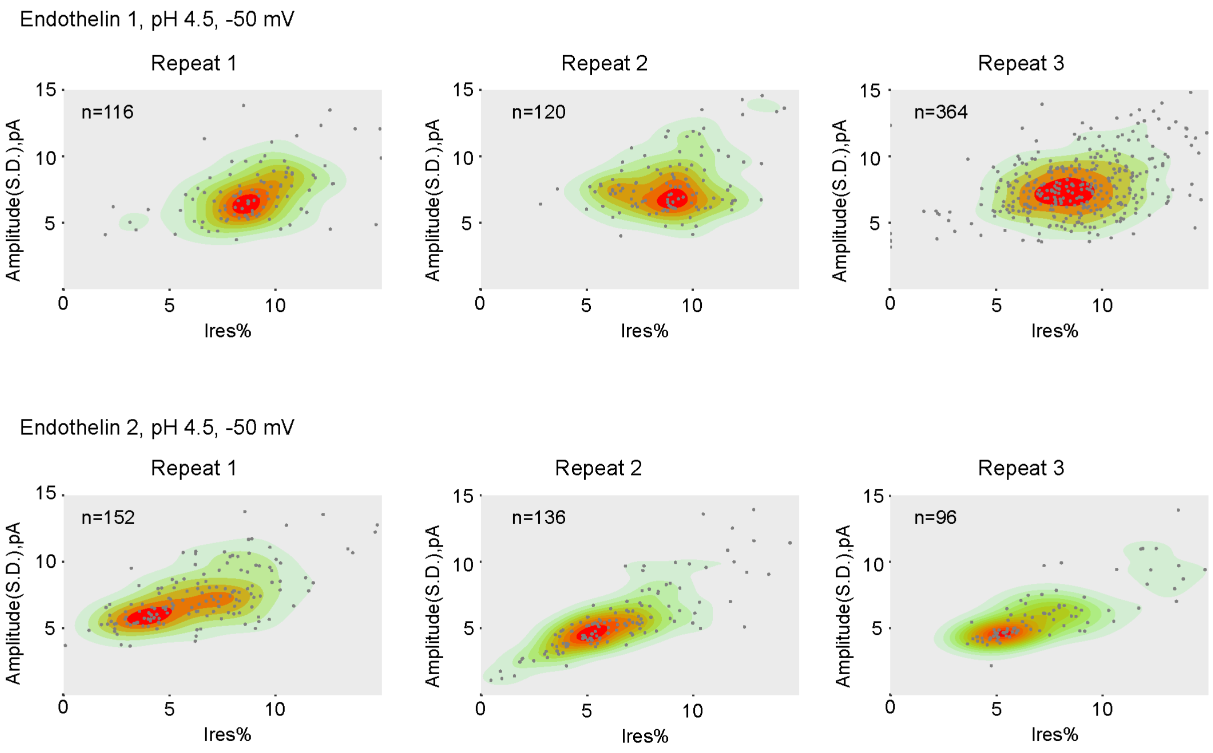

Supplementary Figure 16. $I_{res\%}$ versus the standard deviation of the amplitude of the currents blocks induced by endothelin 1 and 2 with WtFraC at pH 4.5. The buffer was 1 M KCl, 0.1 M citric acid, 180 mM Tris base, pH 4.5 and 200 nM endothelin 1 or 2 µM endothelin 2 was added in to *cis* chamber. Events were recorded with a 50 kHz sampling rate and 10 kHz low-pass Bessel filter. Graphs were created with custom R scripts. Amplitude standard deviations (S.D.) were given by the Clampfit during the single-channel search (Molecular devices).

Supplementary Table 1. Electro-osmotic flux and velocity in WtFraC and ReFraC at pH 4.5 and 7.5

			Electro-osmotic velocity (mm/s)‡		
Pore	pH	Water flux (s^{-1})†	*cis* (R = 2.75 nm)	center (R = 1.0 nm)	*trans* (R = 0.50 nm)
WtFraC	4.5	2.48×10^{9}	3.1	23.6	94.5
	7.5	6.08×10^{9}	7.7	57.9	231.8
ReFraC	4.5	2.08×10^{9}	2.6	19.8	79.2
	7.5	1.37×10^{9}	1.7	13.1	52.3

† As calculated by equation (2) in the main text for 1M KCl at applied potentials of -50 mV and +50 mV for WtFraC and ReFraC, respectively.
‡ Estimate of the direction and magnitude of fluid velocity in the nanopore using the following equation:

$$v = \frac{J_w V_w}{\pi R^2}$$

where v is the velocity of water through the pore, V_w is the volume of water occupied by a single water molecule ($\sim 3.0 \times 10^{-29}$ m^3) and R is the local radius of the pore. J_w is calculated using equation (2) in the main text.

Supplementary Table 2. PROPKA-predicted pKa values for WtFrC.

Residue		Per chain pKa values for WtFraC							
ID	Name	A	B	C	D	E	F	G	H
4	N-term	7.86	7.85	7.85	7.86	7.86	7.85	7.85	7.86
10	ASP	4.36	4.12	4.40	4.13	4.36	4.12	4.40	4.13
17	ASP	2.95	4.01	4.09	3.98	2.95	4.01	4.09	3.98
20	LYS	11.51	10.82	10.76	10.82	11.51	10.82	10.76	10.82
24	GLU	4.66	4.51	4.50	4.54	4.66	4.51	4.50	4.54
30	LYS	10.24	10.24	10.26	10.24	10.24	10.24	10.26	10.24
31	ARG	11.44	11.42	11.45	11.44	11.44	11.42	11.45	11.44
32	LYS	9.74	9.74	9.73	9.74	9.74	9.74	9.73	9.74
38	ASP	3.41	3.40	3.37	3.38	3.41	3.40	3.37	3.38
40	GLU	5.39	5.37	5.39	5.38	5.39	5.37	5.39	5.38
43	LYS	10.05	10.29	10.28	10.27	10.05	10.29	10.28	10.27
51	TYR	11.07	11.06	11.08	11.03	11.07	11.06	11.08	11.03
53	ARG	13.01	12.95	13.06	12.96	13.01	12.95	13.06	12.96
58	ASP	2.70	2.68	2.72	2.70	2.70	2.68	2.72	2.70
63	HIS	3.72	3.61	3.63	3.70	3.72	3.61	3.63	3.70
64	LYS	9.85	9.86	9.86	9.76	9.85	9.86	9.86	9.76
67	HIS	6.27	6.28	6.29	6.28	6.27	6.28	6.29	6.28
69	LYS	10.42	10.42	10.42	10.42	10.42	10.42	10.42	10.42
73	TYR	12.26	12.27	12.35	12.35	12.26	12.27	12.35	12.35
77	LYS	9.03	9.03	9.01	9.02	9.03	9.03	9.01	9.02
79	ARG	12.81	12.86	12.77	12.84	12.81	12.86	12.77	12.84
92	TYR	14.11	14.13	14.22	14.24	14.11	14.13	14.22	14.24
96	ASP	3.03	2.80	2.79	2.87	3.03	2.80	2.80	2.86
108	TYR	10.81	10.82	10.83	10.82	10.81	10.82	10.83	10.82
109	ASP	3.31	3.28	3.32	3.31	3.31	3.28	3.32	3.31
110	TYR	12.50	12.49	12.49	12.52	12.50	12.49	12.49	12.52
113	TYR	10.30	10.30	10.30	10.30	10.30	10.30	10.30	10.30
120	ARG	11.94	11.94	11.94	11.94	11.94	11.94	11.94	11.94
122	TYR	12.33	12.32	12.33	12.31	12.33	12.32	12.33	12.31
123	LYS	10.63	10.58	10.58	10.59	10.63	10.58	10.58	10.59
126	LYS	10.39	10.35	10.35	10.38	10.39	10.35	10.35	10.38
127	ARG	11.45	11.46	11.49	11.48	11.45	11.46	11.49	11.48
129	ASP	4.19	4.17	4.16	4.17	4.18	4.17	4.16	4.17
131	ARG	12.68	12.69	12.69	12.69	12.68	12.69	12.69	12.69
133	TYR	12.48	12.56	12.42	12.57	12.48	12.56	12.42	12.57
134	GLU	3.89	3.86	3.84	3.86	3.88	3.86	3.84	3.86
135	GLU	3.28	3.29	3.31	3.28	3.28	3.29	3.31	3.28
137	TYR	11.44	11.43	11.43	11.46	11.44	11.43	11.43	11.46
138	TYR	10.12	10.12	10.13	10.11	10.12	10.12	10.13	10.11
139	HIS	6.27	6.27	6.27	6.27	6.27	6.27	6.27	6.27
140	ARG	13.78	13.78	13.77	13.78	13.78	13.78	13.77	13.77
144	ARG	12.14	12.14	12.16	12.14	12.14	12.14	12.16	12.14
146	ASP	3.69	3.71	3.64	3.70	3.70	3.71	3.64	3.70
150	HIS	5.63	5.62	5.60	5.64	5.64	5.62	5.61	5.64
152	ARG	11.28	11.28	11.27	11.26	11.28	11.28	11.27	11.26
156	TYR	10.12	10.22	10.22	10.20	10.12	10.22	10.22	10.20
159	LYS	9.98	10.14	9.92	9.98	9.98	10.14	9.92	9.98
161	ARG	11.45	11.45	11.78	11.41	11.45	11.45	11.78	11.41
169	HIS	4.75	4.76	4.78	4.74	4.75	4.76	4.78	4.74
173	GLU	4.41	4.44	4.54	4.40	4.41	4.44	4.54	4.40
175	HIS	6.95	6.94	6.93	6.89	6.95	6.94	6.93	6.89
178	LYS	10.36	10.38	10.38	10.38	10.36	10.38	10.38	10.38
179	C-term	3.35	3.31	3.35	3.35	3.35	3.31	3.35	3.35

Supplementary Table 3. PROPKA-predicted pKa values for ReFraC.

Residue		Per chain pKa values for ReFraC							
ID	Name	ID	Name		ID	Name		ID	Name
4	N+	7.79	7.85	7.81	7.86	7.86	7.85	7.85	7.86
10	ARG	12.25	11.88	11.34	12.01	12.26	11.77	12.10	11.96
17	ASP	3.01	4.07	4.17	4.04	2.96	4.05	4.17	3.96
20	LYS	11.51	10.82	10.77	10.82	11.51	10.82	10.77	10.82
24	GLU	4.66	4.51	4.50	4.54	4.66	4.51	4.50	4.54
30	LYS	10.24	10.24	10.26	10.24	10.24	10.24	10.26	10.24
31	ARG	11.44	11.42	11.45	11.44	11.44	11.42	11.45	11.44
32	LYS	9.74	9.74	9.73	9.74	9.74	9.74	9.73	9.74
38	ASP	3.40	3.40	3.37	3.37	3.41	3.40	3.37	3.38
40	GLU	5.38	5.37	5.39	5.37	5.39	5.36	5.39	5.38
43	LYS	10.05	10.29	10.28	10.27	10.05	10.29	10.28	10.27
51	TYR	11.07	11.06	11.08	11.03	11.07	11.06	11.08	11.03
53	ARG	13.01	12.95	13.06	12.96	13.01	12.95	13.06	12.96
58	ASP	2.70	2.68	2.72	2.70	2.70	2.68	2.72	2.70
63	HIS	3.77	3.67	3.67	3.77	3.77	3.67	3.67	3.77
64	LYS	9.86	9.87	9.87	9.78	9.86	9.87	9.87	9.78
67	HIS	6.27	6.28	6.29	6.28	6.27	6.28	6.29	6.28
69	LYS	10.42	10.42	10.42	10.42	10.42	10.42	10.42	10.42
73	TYR	12.26	12.27	12.35	12.35	12.26	12.27	12.35	12.35
77	LYS	9.03	9.03	9.01	9.02	9.03	9.03	9.01	9.02
79	ARG	12.81	12.86	12.77	12.84	12.81	12.86	12.77	12.84
92	TYR	14.11	14.13	14.22	14.24	14.10	14.13	14.22	14.24
96	ASP	3.03	2.80	2.79	2.86	3.03	2.80	2.79	2.86
108	TYR	10.81	10.82	10.83	10.82	10.81	10.82	10.83	10.82
109	ASP	3.31	3.28	3.32	3.31	3.31	3.28	3.32	3.31
110	TYR	12.50	12.49	12.49	12.52	12.50	12.49	12.49	12.52
113	TYR	10.30	10.30	10.30	10.30	10.30	10.30	10.30	10.30
120	ARG	11.93	11.94	11.94	11.93	11.93	11.94	11.94	11.94
122	TYR	12.33	12.32	12.33	12.31	12.33	12.32	12.33	12.31
123	LYS	10.63	10.58	10.58	10.59	10.63	10.58	10.58	10.59
126	LYS	10.39	10.35	10.35	10.38	10.39	10.35	10.35	10.38
127	ARG	11.46	11.46	11.49	11.48	11.45	11.47	11.48	11.48
129	ASP	4.19	4.17	4.16	4.17	4.18	4.17	4.16	4.17
131	ARG	12.68	12.69	12.69	12.69	12.68	12.69	12.69	12.69
133	TYR	12.48	12.56	12.42	12.57	12.48	12.56	12.42	12.57
134	GLU	3.89	3.86	3.84	3.86	3.88	3.86	3.84	3.86
135	GLU	3.28	3.29	3.31	3.28	3.28	3.29	3.31	3.28
137	TYR	11.44	11.43	11.43	11.46	11.44	11.43	11.43	11.46
138	TYR	10.12	10.12	10.13	10.11	10.12	10.12	10.13	10.11
139	HIS	6.27	6.27	6.27	6.27	6.27	6.27	6.27	6.27
140	ARG	13.78	13.78	13.77	13.77	13.78	13.78	13.77	13.77
144	ARG	12.14	12.14	12.16	12.14	12.14	12.14	12.16	12.14
146	ASP	3.70	3.72	3.64	3.70	3.70	3.71	3.64	3.70
150	HIS	5.64	5.63	5.60	5.65	5.65	5.61	5.60	5.65
152	ARG	11.28	11.28	11.27	11.26	11.28	11.27	11.27	11.26
156	TYR	10.12	10.22	10.22	10.20	10.12	10.22	10.22	10.20
159	GLU	4.92	4.87	4.87	4.92	4.94	4.82	4.84	4.94
161	ARG	11.47	11.50	11.87	11.43	11.47	11.48	11.84	11.45
169	HIS	4.75	4.76	4.78	4.74	4.75	4.76	4.78	4.74
173	GLU	4.40	4.43	4.54	4.38	4.40	4.43	4.53	4.38
175	HIS	6.95	6.95	6.94	6.90	6.95	6.95	6.94	6.90
178	LYS	10.36	10.38	10.38	10.38	10.36	10.38	10.38	10.38
179	C-	3.41	3.38	3.41	3.41	3.42	3.37	3.41	3.42

Supplementary Table 4. I-V curves for WtFraC and ReFraC. †

Voltage(mV)	WtFraC, pH 7.5		WtFraC, pH 4.5		Voltage(mV)	ReFraC, pH 7.5		ReFraC, pH 4.5	
	Current (pA)	S.D.	Current (pA)	S.D.		Current (pA)	S.D.	Current (pA)	S.D.
-200	-544.2	16.2	-381.0	5.4	-100	-142.4	24.1	-152.2	17.6
-180	-508.9	15.7	-349.9	6.6	-80	-112.8	15.0	-117.8	11.1
-160	-467.1	13.0	-319.0	4.3	-60	-86.1	8.7	-87.0	7.9
-140	-422.6	11.3	-286.8	3.9	-40	-59.4	5.2	-56.1	4.8
-120	-375.3	9.7	-252.2	3.4	-20	-30.6	1.7	-23.6	6.6
-100	-324.1	8.1	-215.0	3.3	0	-0.3	0.5	0.4	0.6
-80	-266.9	6.2	-176.1	2.0	20	31.5	1.9	26.5	2.0
-60	-204.3	4.8	-133.8	1.2	40	64.1	2.8	55.0	3.8
-40	-138.3	3.8	-88.9	0.7	60	96.6	3.5	86.6	5.4
-20	-69.0	2.5	-43.4	0.3	80	128.0	3.6	120.8	8.1
0	0.3	0.6	0.4	0.8	100	157.9	3.7	156.7	11.2
20	63.4	1.5	40.2	2.7	120	186.7	3.6	195.3	14.8
40	117.1	7.9	74.3	5.1	140	214.7	4.6	235.0	16.1
60	170.6	19.3	102.4	7.1	160	241.3	5.0	278.7	10.5
80	211.6	36.0	126.3	9.7	180	268.1	4.1	326.0	6.4
100	244.2	50.8	145.6	8.5	200	295.8	4.1	375.8	10.7

† In 1 M KCl solutions at pH 7.5 (15 mM Tris base) and pH 4.5 (0.1 M citric acid, 180 mM Tris base). Each data point represents the average value of at least three repeats. Errors are quoted as standard deviations from 3 repeats.

Supplementary Table 5. Current-voltage (I-V) curves for WtFraC under asymmetric salt conditions.[†]

Voltage (mV)	WtFraC, pH 7.5 1960 mM KCl in *cis*		WtFraC, pH 4.5 1960 mM KCl in *cis*		WtFraC, pH 7.5, 1960 mM KCl in *trans*	
	Current (pA)	S.D.	Current (pA)	S.D.	Current (pA)	S.D.
-29	-179.1	9.9	-96.9	6.7	-34.0	4.2
-28	-174.5	9.7	-93.9	6.6	-31.0	3.9
-27	-169.9	9.4	-91.7	6.0	-28.0	3.7
-26	-165.8	9.6	-88.7	5.3	-25.0	3.4
-25	-161.3	8.9	-86.1	4.9	-22.0	3.2
-24	-156.5	9.0	-82.7	4.5	-19.0	3.0
-23	-152.7	8.2	-80.6	4.3	-16.0	2.9
-22	-148.2	8.2	-77.8	3.9	-12.5	2.5
-21	-144.0	7.7	-75.1	3.5	-9.7	2.2
-20	-139.7	7.3	-72.6	3.4	-6.5	2.0
-19	-135.3	7.1	-68.9	2.9	-3.7	1.8
-18	-131.1	6.8	-67.2	2.8	-0.8	1.7
-17	-126.8	6.3	-64.6	2.7	2.4	1.4
-16	-122.6	5.9	-62.0	2.4	5.4	1.1
-15	-118.4	5.7	-59.4	2.2	8.6	1.1
-14	-114.2	5.4	-56.9	2.2	11.5	1.1
-13	-109.7	7.6	-54.1	2.1	14.5	1.1
-12	-107.0	5.8	-51.7	2.0	17.6	1.0
-11	-102.6	5.6	-49.1	1.9	20.6	1.2
-10	-97.8	5.7	-46.6	2.0	23.8	1.2
-9	-93.7	3.9	-44.0	1.9	27.3	0.4
-8	-89.6	3.6	-41.6	1.9	30.4	0.8
-7	-85.7	3.4	-39.2	2.1	33.7	1.1
-6	-81.7	3.2	-36.7	2.0	36.9	1.2
-5	-77.9	2.8	-34.3	2.0	40.2	1.5
-4	-73.9	2.4	-31.7	2.1	43.1	1.9
-3	-70.1	2.2	-29.5	2.4	46.7	1.7
-2	-65.7	1.2	-27.2	2.4	49.5	2.1
-1	-62.6	1.6	-24.6	2.5	52.8	2.5
0	-58.7	1.4	-22.4	2.4	55.9	2.7
1	-55.1	1.3	-20.0	2.2	59.0	2.9
2	-51.5	1.2	-17.7	2.4	62.1	3.4
3	-47.7	1.1	-15.5	2.5	65.3	3.5
4	-44.3	1.3	-13.3	2.6	68.4	3.8
5	-40.4	1.2	-10.8	3.0	71.8	4.0
6	-36.7	1.4	-8.9	2.7	75.0	4.2
7	-33.4	1.5	-6.7	2.7	77.9	4.5
8	-29.5	2.1	-4.5	2.8	81.0	4.7
9	-25.9	2.8	-2.4	2.8	84.3	5.0
10	-23.1	2.3	-0.3	2.7	87.5	5.4
11	-19.7	2.5	2.0	2.9	87.9	8.0
12	-16.3	2.8	3.9	2.7	93.7	5.8
13	-9.9	8.5	6.0	2.7	96.6	6.1
14	-9.5	3.5	8.0	2.7	100.0	6.1
15	-5.4	3.8	10.0	2.7	104.7	6.6
16	-2.1	4.2	12.2	2.7	107.8	6.9
17	0.8	4.1	14.0	2.7	111.0	7.4
18	4.3	3.4	16.0	2.8	114.1	7.5
19	6.5	4.3	17.9	2.8	117.2	8.1
20	9.4	4.6	19.8	2.7	120.2	8.2
21	12.3	4.9	21.8	2.7	122.8	8.2
22	15.9	5.2	23.7	2.8	125.7	8.1
23	18.7	5.5	25.5	2.8	129.1	9.2
24	20.7	5.9	27.4	2.9	131.9	8.9
25	23.4	6.5	29.2	3.0	134.3	9.2
26	26.2	6.8	30.9	3.0	134.6	13.8
27	28.9	7.2	32.8	3.1	141.7	10.5
28	31.7	7.5	34.1	3.3	146.0	11.8
29	34.4	7.8	36.1	3.1	145.1	14.1
30	37.1	8.0	37.6	3.1	145.8	18.2

[†] The buffer contained either 1960 mM or 467 mM KCl in 15 mM Tris base. Errors are standard deviations obtained with 3 repeats.

Supplementary Table 6. Current-voltage (I-V) curves for ReFraC under asymmetric salt conditions.†

Voltage (mV)	ReFraC pH 7.5 1960 mM KCl in *cis*		ReFraC pH 4.5 1960 mM KCl in *cis*		ReFraC, pH 7.5 1960 mM KCl in *trans*	
	Current (pA)	S.D.	Current (pA)	S.D.	Current (pA)	S.D.
-29	-23.8	5.4	-22.5	3.4	-43.1	4.6
-28	-22.2	5.5	-21.0	3.3	-43.1	2.4
-27	-22.9	3.8	-19.5	3.4	-42.1	2.5
-26	-20.7	4.7	-17.8	3.3	-41.0	2.4
-25	-19.3	4.8	-16.3	3.4	-39.9	2.3
-24	-19.0	3.8	-14.8	3.3	-38.6	2.3
-23	-18.1	3.4	-13.2	3.3	-37.3	2.2
-22	-16.2	3.8	-11.7	3.4	-36.4	2.3
-21	-14.9	3.8	-10.1	3.3	-35.1	2.2
-20	-13.9	3.7	-8.6	3.4	-33.1	1.7
-19	-13.2	3.0	-7.0	3.3	-32.8	2.2
-18	-12.2	2.8	-5.5	3.3	-31.5	2.0
-17	-10.9	2.4	-3.9	3.4	-30.6	2.1
-16	-9.5	2.7	-2.3	3.4	-29.1	1.9
-15	-8.3	2.4	-0.7	3.4	-27.9	1.9
-14	-7.4	2.1	0.9	3.5	-26.7	2.0
-13	-5.9	1.9	2.5	3.5	-25.7	2.0
-12	-4.7	1.8	4.1	3.5	-24.4	1.9
-11	-3.8	1.5	5.7	3.5	-23.2	1.9
-10	-2.9	1.5	7.3	3.5	-22.0	1.8
-9	-1.7	1.1	8.9	3.5	-20.7	1.8
-8	-0.3	1.5	10.6	3.6	-19.4	1.7
-7	1.8	1.2	12.3	3.6	-18.3	1.7
-6	2.8	0.8	13.9	3.7	-17.0	1.6
-5	3.8	0.6	15.6	3.8	-15.7	1.5
-4	5.2	0.4	17.3	3.8	-14.6	1.5
-3	6.5	0.2	19.0	3.9	-13.2	1.4
-2	8.1	0.2	20.7	3.9	-11.5	1.4
-1	9.4	0.2	22.4	4.1	-10.6	1.3
0	10.7	0.3	24.1	4.1	-9.5	1.3
1	12.0	0.4	25.9	4.2	-8.2	1.3
2	12.4	1.6	27.7	4.2	-6.9	1.2
3	13.5	2.0	29.4	4.3	-5.7	1.2
4	15.3	2.4	31.2	4.4	-4.4	1.2
5	17.6	1.1	33.0	4.6	-3.2	1.1
6	19.0	1.4	34.8	4.7	-1.9	1.1
7	19.8	2.6	36.6	4.8	-0.6	1.1
8	21.5	2.2	38.5	4.9	0.7	1.1
9	23.8	1.3	40.3	5.0	2.1	1.1
10	25.2	1.5	42.2	5.1	3.4	1.2
11	26.6	1.8	44.0	5.3	4.8	1.2
12	28.3	1.8	45.7	5.9	6.0	1.3
13	28.6	3.5	48.0	5.6	7.3	1.5
14	30.7	2.7	50.0	5.8	8.7	1.5
15	32.3	2.7	52.0	6.0	10.0	1.6
16	31.9	5.8	54.0	6.1	11.2	1.6
17	34.9	3.4	55.5	7.0	12.6	2.0
18	36.2	3.7	57.3	7.0	13.7	1.7
19	37.9	3.7	59.5	7.4	14.8	1.5
20	39.5	3.9	61.1	7.7	16.3	1.9
21	39.8	5.1	63.3	7.6	17.7	2.1
22	42.0	3.4	65.5	7.9	19.2	2.6
23	43.7	3.7	67.5	8.3	20.4	2.9
24	43.8	5.2	69.6	8.4	21.9	2.8
25	43.9	8.0	71.7	8.6	23.1	3.1
26	46.3	6.8	73.9	8.8	24.5	3.2
27	49.5	5.7	75.7	8.9	25.8	3.3
28	51.4	5.0	78.1	9.3	27.1	3.4
29	52.6	6.1	80.3	9.5	28.4	3.7
30	54.1	6.0	82.5	9.7	29.8	3.9

† The solution contained either 1960 mM or 467 mM KCl in 15 mM Tris base. Errors are standard deviations obtained with 3 repeats.

Supplementary Table 7. Characterization of peptide and protein biomarkers at different voltages with WtFraC.†

Voltage (mV)	Dwell time (ms)	Ires%	Capture frequency (s^{-1} μM^{-1})	Voltage (mV)	Dwell time (ms)	Ires%	Capture frequency (s^{-1} μM^{-1})
**Chymotrypsin**				**Human EGF**			
**-110**	8.9±7.8	23.4±0.6	44.1±4.8	**-50**	17.1±3.0	2.0±0.2	2.6±1.4
**-130**	28.3±16.5	19.7±0.4	63.6±4.7	**-70**	45.7±15.0	1.6±0.3	7.8±4.7
**-150**	134.4±95.6	17.3±0.3	79.8±4.7	**-80**	77.3±28.9	1.4±0.3	13.1±8.2
**-170**	279.0±119.0	15.5±0.2	91.8±19.5	**-90**	82.7±27.3	1.2±0.3	20.8±12.8
**-190**	1086±547	14.0±0.5	99.8±23.8	**-100**	68.4±13.6	1.5±0.3	29.5±16.7
				**-140**	14.6±0.5	1.4±0.2	82.9±40.8
**β2-microglobulin**				**Endothelin 1**			
**-50**	376.6±124.4	0.1±0.8	1.5±0.8	**-10**	18.8±1.2	4.0±0.3	1.5±0.2
**-70**	249.3±108.8	0.1±0.4	3.3±1.5	**-20**	20.7±1.9	6.9±0.2	2.2±0.5
**-90**	157.7±76.4	0±0.1	5.4±1.7	**-30**	11.9±2.2	7.9±0.1	3.2±0.6
**-110**	98.1±49.6	0±0.2	6.7±1.9	**-40**	8.3±3.2	8.2±0.5	3.9±1.2
				**-50**	3.7±0.5	9.1±0.1	5.8±0.7
**Angiotensin I**							
**-10**	0.22±0.02	42.5±0.4	19.1±3.9				
**-20**	0.17±0.02	43.6±0.1	26.5±2.2				
**-30**	0.15±0.04	43.4±0.9	27.2±2.5				

† Chymotrypsin was analysed in pH 7.5 buffered solutions (1 M KCl, 15 mM Tris base), while all other four biomarkers were analysed in pH 4.5 buffered solutions containing 1M KCl, 0.1 M citric acid, 180 mM Tris base. Experiments were repeated three times and at least 1000 events were captured, except for chymotrypsin at -190 mV where 621 events were collected. Errors are quoted as standard deviations from 3 repeats.
