# Supplementary material for: Electro-osmotic capture and ionic discrimination of peptide and protein biomarkers with FraC nanopores
Source: Nat Commun. 2017 Oct 16;8:935. doi: 10.1038/s41467-017-01006-4 (PMC5715100; doi:10.1038/s41467-017-01006-4)
Supplement: Supplementary file 2 — Peer Review File [file 41467_2017_1006_MOESM2_ESM.pdf]

Reviewers' comments:

Reviewer #1 (Remarks to the Author):

see attached file

Reviewer #2 (Remarks to the Author):

In this manuscript titled "Electro-osmotic Capture and Ionic Discrimination of Peptide and Protein Biomarkers with FraC Nanopores", the authors report a FraC nanopore-based approach for the identification and distinguishing of homologous peptides and proteins. FraC was engineered to generate strong EOF to attract the proteins into the pore lumen. Using EOF as the driving force makes it a versatile method for analyzing proteins carrying different surface charge. Five protein analytes ranging from 1.3 kDa to 25 kDa were investigated. They generated distinct ionic current blockades for identification with FraC. Particularly FraC was shown to resolve endothelin proteins with just a single amino acid variation. This is a well conducted work, where a new biological nanopore is introduced for protein identification with high resolution. The manuscript is clearly written and enjoyable to read. I recommend the publication of this work in Nature communication with minor revision.
The minor issues are the following:
Page 5 line 102: "Since the constriction of ClyA is lined with aspartic acid" the ClyA should be FraC. In the SI, page 17 line 220: The same mistake
Page 5 line 105: "Simultaneously, a less negative endothelin 1 would also migrate more easily towards the trans electrode under". Simultaneously? It is unclear to me. Please reword this sentence.
Page 18, figure 4b: please highlight the residue F14 of ET-1 and E14 of ET-2 to make it easier for readers to spot the difference between the two peptides.

Reviewer #3 (Remarks to the Author):

This manuscript by Huang et al explores the use of a biological nanopore protein, FraC, for the detection of seven peptide and small protein biomarkers. The authors establish experimental conditions that take advantage of electroosmotic flow to move all the analytes into the conically shaped constriction of the pore leading to biomarker-specific current blockades and residence times. The most compelling result with regard to biomarker analysis is that the 21 amino acid peptide endothelin 1 can be distinguished from endothelin 2, which differs only by replacement of two amino acids (not one as stated by the authors). This is an impressive and inspiring result in the sense that even small differences are detectable. The authors are, however, also demonstrating that in the case of another peptide, angiotensin I with 10 amino acids, cannot be distinguished from angiotensin II, which is missing the last two amino acids of angiotensin I. The authors also present interesting insight into the electrostatics and electroosmotic flow in both wild type FraC pores (WtFraC) with a predominantly negatively charged pore constriction and in a mutant (ReFraC) with a predominantly positively charged pore constriction. The authors show that these two pores generate electroosmotic flow in opposing directions if the polarity of the applied potential difference is kept constant. Finally, the authors conducted a thorough characterization of the behavior of the different biomarkers in the vestibule of the pore. Based on residence time as a function of applied potential difference, the authors present evidence for two different cases: A first case in which small proteins and large peptides exit the pore from the same side as they entered irrespective of the magnitude of the applied potential and a second case, in which small peptides translocate through the pores at sufficiently high potential differences. Importantly, WtFraC induced electroosmotic flow from the large vestibule side of

the pore to its narrow constriction even at pH 4.5. At this pH the biomarkers were either positively charged or only slightly negatively charged, such that EOF dominated and delivered all biomarkers into the pore for detection.

Altogether this manuscript presents interesting results with substantial data supporting the claims and conclusions. The data is of high quality, the study is very well done and convincing. The figures, structures, and cartoons are outstanding in clarity and appeal. The work is a significant contribution to the analysis of peptides, small proteins, and biomarkers to the growing nanopore field.

Comments:

The last sentence of the abstract is unclear, confusing, and misleading. Only a subset of very small proteins or peptides examined here translocated through the pores, others didn't. Reading the entire manuscript one is not left with the sense that a "general rationale" is being presented. The authors either have to explain better what they mean, make more convincing arguments for this statement, or simply rewrite the concluding statement altogether.

The sentence ending in line 51 needs a reference(s).

Line 62 to 64: It appears that if the authors would have chosen a slightly more acidic pH, such as pH 4.0, the condition that they say can not be achieved might actually have been achieved -- unless the authors know that at pH 4.0 the direction of WtFraC is reversed compared to pH 4.5.

For a high profile journal such as Nature Communications, the "writing" of this manuscript could be improved, both on the sentence level and the "storytelling" level. Early in the results section the text is hard to read because it presents detail that appears at times redundant with the figures or could possibly be put better in context rather than stating quantitative values (for instance in lines 124 to 136 as well as on the previous page). For this reason, the manuscript seems to lose the plot at times.

In both the introduction and the discussion, there seems to be a fixation on protein sequencing that doesn't seem particularly relevant, given that this paper makes no effort to sequence the analytes. Possibly move the speculation about FraC possible usefulness for protein sequencing towards the end of the discussion and discuss better why the authors feel that it may be an excellent pore for that (which it may well be but which will have to be demonstrated).

The authors should discuss whether the concentrations of biomarkers that they demonstrated for their experiments are in the relevant physiological and pathophysiological range for these biomarkers. The authors should also touch on possible anticipated challenges when applying this approach to biomarker detection in relevant biofluids.

Line 126: What do the authors mean by "...the blockades...became homogeneous..."?

The large body of interesting results presented in Figure 3 should be discussed in more detail. For instance, the results on kon rates for different peptides are scarcely discussed and put into context. What is their relevance?

The "reversal potential" label above Fig. 2b is confusing, as "reversal potential" refers only to the x-intercept of those curves.

Just a suggestion: Wouldn't the graphs in Fig. 3 better convey the point that dwell time, Ires%, and kon are potential fingerprinting parameters if they all had the same axes, even if it's worse from a

data presentation perspective?

First sentence in the conclusion: Mention that detection of single biomarkers in pure buffer solutions is possible and shows differences between peptides and in a binary mixture entdothelin I and II could be distinguished rather than a general statement that may imply that these biomarkers may be distinguishable in a mixture of all of them (or leave the sentence as is and show this capability).

The last sentence in the conclusion appears too strong "...it is likely...". Consider, "...it may be possible..."

Referee report manuscript NCOMMS-17-08137

The manuscript "Electro-Osmotic Capture and Ionic Discrimination of Peptide and Protein Biomarkers with FraC Nanopores" by Huang et al describes the detection of peptide and protein biomarkers from 25 kDa down to 1.2 kDa using protein channels, Fragaceatoxin C (Frac C) and a modified recombinant nanopore (D10R, K159E FraC (ReFraC) with arginine residues at the constriction. The capture of peptides and proteins is well controlled by the electro-osmotic flow according to the electrostatic distribution inside the 2 channels and the ion selectivity of the nanopores as a function of pH. The characterization of each protein and peptide as a biomarker model is shown with a specific electrical signature of the dwell time and by the normalized current blockade (Ires%, defined as (IB/IO)*100). Peptides, Endothelin 1 and 2 that differ just by one tryptophan residue are detected by current traces.

The manuscript is interesting to scientists in the nanopore and protein field but also to people in confined medium dynamics field. In the future, the possibility to detect few copies of protein biomarkers in body fluids is a real challenge but also a societal issue for medical biology and biotechnology. The work of Huang et al could be the start of this challenge and societal issue.

The manuscript is clearly written. Experiments, data analysis, statistical analysis and simulations are well performed. The experiments could be reproduced.

However, state of the art, novelty according to previous papers, data discussion and new experiments recommendations should be addressed.

Recommendation: reconsider after major revisions

-State of the art and Novelty

Previous studies have been performed, by several groups, to detect a wide range of proteins of different molecular masses, with glass nanopores from 12 kDa to 480 kDa, (Steinbock et al, Nanoscale, 2014; Li et al, ACS Nano, 2016) or with solid-state nanopores coated with lipids (Yusko et al, 2016, Nat Nanotech). The pH variation on the net charge of the protein was studied by showing a change in the translocation direction (Steinbock et al, Nanoscale, 2014). Recently, numerous proteins have been detected and their conformational changes with solid-state

nanopores with a balance of electro-osmotic flow and electrophoresis (Waduge et al, ACS Nano, 2017).

Could you please had these references and introduce the main results.

You must discuss deeply the significant input of your work according to the previous papers (what is new, discrimination power of protein in molecular masses, threshold concentration of detection, applicable for other proteins or peptides, characterization of single amino-acid mutation….).

L67. “Nanopores with a large diameter revealed that globular proteins might translocate too quickly ..if the diffusion of the protein … ionic current blockades can be used to identify proteins”.

Could you add the reference “Oukhaled et al, 2011, ACS Nano” and “Balme et al, 2016, Langmuir”. In the presence of interactions between the nanopore surface (solid-state) and proteins, the dynamic is slowed down and the proteins can be detected (MBP, avidin, lysozyme, and IgG).

Results and discussion

- Peptide and protein capture with FraC nanopores

Fig1. “Representative traces induced by 1 μM 326 endothelin 1 (b) and 200 nM chymotrypsin (c).

The concentrations used in these experiments are very high in comparison to the goal of this manuscript, detection of biomarkers; it means few dozen or few hundred of molecules. What is the threshold concentration in your sep-up?

It will be very interesting to add a titration curve for one or 2 proteins, event frequency as a function of concentration.

- Ion selectivity and electrostatic potential of FraC nanopores.

L140. “To gain a better insight into the influence of pH on the electrostatic environment inside FraC 141 nanopores, we used the Adaptive Poisson-Boltzmann Solver (APBS)46 and a modified version 142 of the PDB2PQR software47”

“The resulting PQR files were used for solving the Poisson-Boltzmann equation with APBS” (sup material)
For a journal of broad audience, you must introduce clearly the physical concept, the equation and methodology used.
I have several questions:
*What is the geometry used to solve the Poisson-Boltzmann equation?
*Why Adaptive Poisson-Boltzmann solver?
*What is the electroosmotic velocity through FraC nanopores?
*Is there a limit in protein size (KDa) where the EOF cannot overcome the electrophoretic force?
*What is the general condition for a protein to be captured by EOF into a protein channel?

-Near-isomeric polypeptide discrimination and Folded proteins may translocate FraC nanopores by deforming the transmembrane region of the nanopore.
*Figure 3. What are the fit used for the dwell times as a function of V?
“Together, these findings suggest that the transmembrane region of the nanopore deforms during the translocation of folded β2-microgobulin molecules”
*If the transmembrane region of the nanopore deforms during the translocation of folded β2-microgobulin, why does the Ires remain constant as a function of V? A deformation could alter the pore diameter and the ratio of protein/pore volume.
*What is the mechanism of channel deformation?

L261. Interestingly, the Ires% of endothelin 1 increased with the applied potential, suggesting that this polypeptide may be stretched by the increased EOF through the nanopore.
-Could you please discuss why the polypeptide is completely stretched inside the channel?
-Could you please cite papers already showing this phenomenon with protein or polypeptide chains that are stretched under the applied voltage (Cressiot et al, 2012, ACS Nano; Freedman et al, 2013, Sci Rep.).

-New experiments to perform

*The Protein-channel Interactions (electrostatic…) are crucial for the capture and for the detection of peptides and proteins within the nanopore. I recommend evaluating the kinetic rate constant and the constant of association/dissociation for the different protein biomarkers.

*I recommend performing a new experiment with a mix of different proteins in order to show the power of discrimination of this protein sensor under EOF driving force.

Reviewer #1 (Remarks to the Author):
The manuscript "Electro-Osmotic Capture and Ionic Discrimination of Peptide and Protein Biomarkers with FraC Nanopores" by Huang et al describes the detection of peptide and protein biomarkers from 25 kDa down to 1.2 kDa using protein channels, Fragaceatoxin C (Frac C) and a modified recombinant nanopore (D10R, K159E FraC (ReFraC) with arginine residues at the constriction. The capture of peptides and proteins is well controlled by the electro-osmotic flow according to the electrostatic distribution inside the 2 channels and the ion selectivity of the nanopores as a function of pH. The characterization of each protein and peptide as a biomarker model is shown with a specific electrical signature of the dwell time and by the normalized current blockade (Ires%, defined as (IB/IO)*100). Peptides, Endothelin 1 and 2 that differ just by one tryptophan residue are detected by current traces. The manuscript is interesting to scientists in the nanopore and protein field but also to people in confined medium dynamics field. In the future, the possibility to detect few copies of protein biomarkers in body fluids is a real challenge but also a societal issue for medical biology and biotechnology. The work of Huang et al could be the start of this challenge and societal issue. The manuscript is clearly written. Experiments, data analysis, statistical analysis and simulations are well performed. The experiments could be reproduced. However, state of the art, novelty according to previous papers, data discussion and new experiments recommendations should be addressed.
Recommendation: reconsider after major revisions

We thank the reviewer for the positive assessment.

-State of the art and Novelty
Previous studies have been performed, by several groups, to detect a wide range of proteins of different molecular masses, with glass nanopores from 12 kDa to 480 kDa, (Steinbock et al, Nanoscale, 2014; Li et al, ACS Nano, 2016) or with solid-state nanopores coated with lipids (Yusko et al, 2016, Nat Nanotech). The pH variation on the net charge of the protein was studied by showing a change in the translocation direction (Steinbock et al, Nanoscale, 2014). Recently, numerous proteins have been detected and their conformational changes with solid-state nanopores with a balance of electro-osmotic flow and electrophoresis (Waduge et al, ACS Nano, 2017).
Could you please had these references and introduce the main results.
We added the references suggested by the reviewers. We assume Li et al, ACS Nano 2016 refers to Li et al., ACS Nano 2013 (PMID: 23607870)

You must discuss deeply the significant input of your work according to the previous papers (what is new, discrimination power of protein in molecular masses, threshold concentration of detection, applicable for other proteins or peptides, characterization of single amino-acid mutation....).
We have added an extensive discussion on the point raised by the reviewer throughout the manuscript.
In the introduction we have reviewed the achievements of solid-state nanopores and glass pipettes, which sampled large proteins. We also have referred to other work where small differences in proteins are identified. In the discussion we have examined the issue of low-abundance protein and the limits of this technology.
It should be noticed, however, that the main focus of this manuscript lies in the ability of nanopores to recognize different biomarkers especially small polypeptides (<12 kD) and in the engineering of the nanopore to capture and translocate the polypeptide analytes. We have therefore extensively discussed the literature regarding these points.

L67. "Nanopores with a large diameter revealed that globular proteins might translocate too quickly. if the diffusion of the protein ... ionic current blockades can be used to identify proteins". Could you add the reference "Oukhaled et al, 2011, ACS Nano" and "Balme et al, 2016, Langmuir". In the presence of interactions between the nanopore surface (solid-state) and proteins, the dynamic is slowed down and the proteins can be detected (MBP, avidin, lysozyme, and IgG).
We added both references and comments in the text.

Results and discussion
- Peptide and protein capture with FraC nanopores
Fig1. "Representative traces induced by 1 µM 326 endothelin 1 (b) and 200 nM chymotrypsin(c).
The concentrations used in these experiments are very high in comparison to the goal of this manuscript, detection of biomarkers; it means few dozen or few hundred of molecules. What is the threshold concentration in your sep-up?
It will be very interesting to add a titration curve for one or 2 proteins, event frequency as a function of concentration.
We realize that the concentration of many biomarkers in biological fluids is lower than the concentrations sampled here. We did not initially discuss this issue because the goal of this manuscript is to demonstrate the potential of the FraC nanopores to discriminate among proteinaceous biomarkers. To answer the reviewer, in the final implementation, it is likely that targeted biomarkers will be either purified or enriched prior analysis as it is standard with most proteomic techniques. As for the threshold concentration for detection, the reader can calculate it based on the capture frequency of every biomarker, which is given in Figure 3. For example, assuming that at least 100 events are recorded for each analyte, and the experiment can be

performed over 1000 seconds (about 15 minutes), the threshold corresponds to a frequency of 0.1 events per seconds. From the graph in Fig. 3 the concentration threshold limit varies from ~10 nM (endothelin 1) to 1 nM (EGF).

However, we think the point raised by the review is valid and we have now discussed this issue in the manuscript (second paragraph, discussion).

- Ion selectivity and electrostatic potential of FraC nanopores.
L140. "To gain a better insight into the influence of pH on the electrostatic environment inside FraC 141 nanopores, we used the Adaptive Poisson-Boltzmann Solver (APBS)46 and a modified version 142 of the PDB2PQR software47"
"The resulting PQR files were used for solving the Poisson-Boltzmann equation with APBS" (sup material)
For a journal of broad audience, you must introduce clearly the physical concept, the equation and methodology used.
I have several questions:
*What is the geometry used to solve the Poisson-Boltzmann equation?
APBS solves the PB equation in the following form:

$$-\nabla \cdot [\epsilon(\boldsymbol{r})\nabla\phi(\boldsymbol{r})] + \kappa(\boldsymbol{r})^{-2}\sinh(\phi(\boldsymbol{r})) = \rho^{f}(\boldsymbol{r})$$

A lipid bilayer mimic was added in these maps in the form of a dielectric slab ($\epsilon = 2$), inaccessible for mobile ions, with the C-program 'draw_membrane2' (included as a tool with APBS). This software reads in dielectric, ion-accessibility and charge maps produced by APBS and modifies them to include a solid dielectric slab with controllable thickness, position and central opening to accommodate for transmembrane pores. The domain occupied by the lipid bilayer was given a dielectric constant of 2 and an ion-accessibility of 0.
The geometry for the dielectric constant is based on the molecular surface of the solute, where the bulk electrolyte region ($\epsilon = 80$) is defined as the union of spheres with a given solvent radius ($R = 1.4$ Å) that do not overlap with any solute atoms. The complement of this domain is assigned the solute dielectric constant ($\epsilon = 10$). A similar approach is used to determine the ion-accessibility, with the difference that the radii of all solute atoms are increased with the given radius mobile ion radius ($R = 2.0$ Å). The mobile ions are assigned full accessibility ($\kappa = 1$) in the bulk electrolyte and no accessibility ($\kappa = 0$) with the solute domain defined for the dielectric constant. To reduce sensitivity to the grid setup, all grid values are harmonically averaged over 9 adjacent grid points (doi: 10.1002/(SICI)1096-987X(19970130)18:2<268::AID-JCC11>3.0.CO;2-E). Atomic partial charges are mapped onto nearest- and next-nearest-neighbor grid points by cubic B-spline discretization ($\rho^{f}(\boldsymbol{r})$).

We have specified these issues in *methods*.

*Why Adaptive Poisson-Boltzmann solver?
The purpose of the simulations in this work is to gain a qualitative insight into the different electrostatic potential distributions for WtFraC and ReFraC at pH 4.5 and 7.5. We opted for APBS to tackle this problem due to its robustness, speed and ease of use. While calculating the electrostatics using explicit solvent Poisson-Boltzmann methodology would likely be more accurate, the increased complexity of the model would result in a much higher computational cost.

*What is the electroosmotic velocity through FraC nanopores?
We have calculated the electroosmotic velocities within the nanopore and added to manuscript (Supplementary Table 1).

*Is there a limit in protein size (KDa) where the EOF cannot overcome the electrophoretic force?
For larger proteins, it seems the electroosmotic force has a more dominant role than the electrophoretic force. However, protein surface charges and the mass/charge of a protein likely play also a role in their capture which is why there is probably no universal protein size limit. Within the tested biomarkers, selected over a wide range of sizes, we have found no limitation yet. However, it is not likely that highly negatively charged proteins will translocate against the electrophoretic force. The introduction of unnatural amino acids at the nanopore constriction might be necessary to induce an electro-osmotic flow at pH lower than 4.0. Under such pH the negatively charged amino acids will become neutral and the EOF will likely drive translocation across the nanopore.
The force exerted by an electrical field on a charged spherical particle can be approximated by $F_{ep} = q\boldsymbol{E} \sim q\frac{V}{l}$, where $\boldsymbol{F}_{ep}$ is the electric force, q the charge of the particle and $\boldsymbol{E}$ the electrical field in the pore, defined as the applied bias voltage (V) divided by the length of the pore (l). At 100 mV bias, the voltage drop in the 10 nm long channel of a FraC nanopore would result in an electrical field of ~10 mV/nm, yielding a force of ~1.6 pN per elementary charge. Note that this is only a rough estimate, as it does not consider the charge and shape of the nanopore. Further, this should also be considered as an upper limit, since work with single stranded DNA and αHL nanopores revealed forces on the order of ~0.3 pN per charge under 100 mV (*i.e.* ~10 pN on ~ 20 bases at +160 mV, DOI: 10.1088/0957-4484/26/8/084002) most likely due to the screening by counter ions (doi: DOI: 10.1103/PhysRevLett.85.3057). The maximum force which can be exerted by the electroosmotic flow can be approximated using Stokes' law: $F_{eo} = 6\pi\eta r v$, where F_{eo} is the electro-osmotic force, η is the dynamic viscosity of water (9.0×10^{-4} Ns/m^2 at 25°C), r is the radius of the particle and v is the velocity of the solvent. For endothelin-1 ($r \approx 1.2\ nm$) and chymotrypsin ($r \approx 2.4\ nm$), assuming a water velocity of 100 mm/s (*e.g.* doi:10.1007/s10404-017-1928-1, and WtFraC at pH 7.5 and -100 mV bias voltage, **Supplementary Table 1**), this results in forces of 2.0 and 4.0 pN, respectively. Thus, considering these rough estimates, using WtFraC nanopores, the F_{eo} is stronger than the F_{ep} for small polypeptides such as endothelin 1 when they contain fewer than

about seven negative charges (out of the 21 amino acids). However, these are just rough approximation that have large uncertainty given we do not know the exact electric field and F_{ep} inside the nanopore, the charge of the residues (especially at lower pH), the strength of the EOF and the electrostatic interactions between the pore and the peptide. Therefore, we have not included these considerations in the manuscript.

*What is the general condition for a protein to be captured by EOF into a protein channel?
Using WtFraC at pH 4.5, we demonstrated capture of small proteins (β2-microglobulin, EGF) and peptides (endothelin 1, 2 and angiotensin 1). A larger protein (chymotrypsin) was less efficiently captured under this condition (Fig. 1c), presumably due to the reduced EOF of WtFraC at pH 4.5. Thus, we presume that smaller proteins and peptides can be captured under this condition in general (WtFraC, pH 4.5) where the charges are moderated but EOF is still relatively strong; while larger proteins may enter WtFraC at low potentials only at higher pH with stronger EOF.
The issue of electrophoretic versus electroosmotic capture of polypeptides is now discussed in the main text

-Near-isomeric polypeptide discrimination and Folded proteins may translocate FraC nanopores by deforming the transmembrane region of the nanopore.
*Figure 3. What are the fit used for the dwell times as a function of V?
In the initial manuscript we used a b-spline (Origin), as it was specified in the figure legend. This is because linear and exponential fits could not be employed for all figures. Following the recommendation of the reviewer, we discussed more about the entry and exit of polypeptides in the revised main text and amended the fitting of the dwell times over voltage where possible. In the new figure, exponential fitting is used for the dwell time dependency on voltage with the exception for EGF. In fact, the dwell time dependency on voltage of EGF can be fitted into exponential function at the voltages lower than -90 mV or higher than -90 mV. However, we just used b-spline (Origin) to connect the plots in EGF.

"Together, these findings suggest that the transmembrane region of the nanopore deforms during the translocation of folded β2-microgobulin molecules"
*If the transmembrane region of the nanopore deforms during the translocation of folded β2-microgobulin, why does the Ires remain constant as a function of V? A deformation could alter the pore diameter and the ratio of protein/pore volume.
The transmembrane region may deform to the minimal size required to permit the translocation of β2-microgobulin. Thus, β2-microgobulin may obstruct fully at any stage the ionic current flowing through FraC as we observed essentially no residual current at any voltage.

*What is the mechanism of channel deformation?
The transmembrane region of FraC is made by α-helices at the N-terminus of the protein. We think that such α-helices are structurally more flexible than the β-sheet loops of other nanopores, which in turn allows the (reversible) deformation of the nanopore. How this mechanism is just a speculation.

L261. Interestingly, the Ires% of endothelin 1 increased with the applied potential, suggesting that this polypeptide may be stretched by the increased EOF through the nanopore.
-Could you please discuss why the polypeptide is completely stretched inside the channel?
We assume that polypeptides can be stretched by the electroosmotic flow (from *cis* to *trans*). When an analyte is negatively charged and the electrophoretic force will further stretch the polypeptide by pulling in the opposite direction (from *trans* to *cis* under negative applied potential). However, the stretching of the polypeptide was inferred only by the increased Ires as the potential was increased. We currently do not know if polypeptides are fully stretched inside the pore.

-Could you please cite papers already showing this phenomenon with protein or polypeptide chains that are stretched under the applied voltage (Cressiot et al, 2012, ACS Nano; Freedman et al, 2013, Sci Rep.).
We added these references.

-New experiments to perform
*The Protein-channel Interactions (electrostatic…) are crucial for the capture and for the detection of peptides and proteins within the nanopore. I recommend evaluating the kinetic rate constant and the constant of association/dissociation for the different protein biomarkers.
We have added a comment on the voltage dependency of the capture rates. The dwell times were already discussed in the main text

*I recommend performing a new experiment with a mix of different proteins in order to show the power of discrimination of this protein sensor under EOF driving force.
We did this, separating β2-microglobulin; EGF and endothelin 1 from each other (See new Figure 4 in main text)

Reviewer #2 (Remarks to the Author):

In this manuscript titled "Electro-osmotic Capture and Ionic Discrimination of Peptide and Protein Biomarkers with FraC Nanopores", the authors report a FraC nanopore-based approach for the identification and distinguishing of homologous peptides and proteins. FraC was engineered to generate strong EOF to attract the proteins into the pore lumen. Using EOF as the driving force makes it a versatile method for analyzing proteins carrying different surface charge. Five protein analytes ranging from 1.3 kDa to 25 kDa were investigated. They generated distinct ionic current blockades for identification with FraC. Particularly FraC was shown to resolve endothelin proteins with just a single amino acid variation. This is a well conducted work, where a new biological nanopore is introduced for protein identification with high resolution. The manuscript is clearly written and enjoyable to read. I recommend the publication of this work in Nature communication with minor revision.

We thank the reviewer for the positive assessment.

The minor issues are the following:

Page 5 line 102: "Since the constriction of ClyA is lined with aspartic acid" the ClyA should be FraC.

We fixed this.

In the SI, page 17 line 220: The same mistake

We fixed this.

Page 5 line 105: "Simultaneously, a less negative endothelin 1 would also migrate more easily towards the trans electrode under". Simultaneously? It is unclear to me. Please reword this sentence.

Endothelin 1 becomes less negatively charged under acidic conditions. Thus, acidic pH decreases the electrophoretic force that opposed translocation (at negative applied transmembrane potential). At the same time (hence simultaneously) the negative charge at the constriction is reduced and the energy barrier for negative charges passing the constriction is lowered.

Page 18, figure 4b: please highlight the residue F14 of ET-1 and E14 of ET-2 to make it easier for readers to spot the difference between the two peptides.

We have highlighted the residue in question.

Reviewer #3 (Remarks to the Author):

This manuscript by Huang et al explores the use of a biological nanopore protein, FraC, for the detection of seven peptide and small protein biomarkers. The authors establish experimental conditions that take advantage of electroosmotic flow to move all the analytes into the conically shaped constriction of the pore leading to biomarker-specific current blockades and residence times. The most compelling result with regard to biomarker analysis is that the 21 amino acid peptide endothelin 1 can be distinguished from endothelin 2, which differs only by replacement of two amino acids (not one as stated by the authors). This is an impressive and inspiring result in the sense that even small differences are detectable. The authors are, however, also demonstrating that in the case of another peptide, angiotensin I with 10 amino acids, cannot be distinguished from angiotensin II, which is missing the last two amino acids of angiotensin I. The authors also present interesting insight into the electrostatics and electroosmotic flow in both wild type FraC pores (WtFraC) with a predominantly negatively charged pore constriction and in a mutant (ReFraC) with a predominantly positively charged pore constriction. The authors show that these two pores generate electroosmotic flow in opposing directions if the polarity of the applied potential difference is kept constant. Finally, the authors conducted a thorough characterization of the behavior of the different biomarkers in the vestibule of the pore. Based on residence time as a function of applied potential difference, the authors present evidence for two different cases: A first case in which small proteins and large peptides exit the pore from the same side as they entered irrespective of the magnitude of the applied potential and a second case, in which small peptides translocate through the pores at sufficiently high potential differences. Importantly, WtFraC induced electroosmotic flow from the large vestibule side of the pore to its narrow constriction even at pH 4.5. At this pH the biomarkers were either positively charged or only slightly negatively charged, such that EOF dominated and delivered all biomarkers into the pore for detection.

Altogether this manuscript presents interesting results with substantial data supporting the claims and conclusions. The data is of high quality, the study is very well done and convincing. The figures, structures, and cartoons are outstanding in clarity and appeal. The work is a significant contribution to the analysis of peptides, small proteins, and biomarkers to the growing nanopore field.

We thank the reviewer for the overall positive assessment.

Comments:

The last sentence of the abstract is unclear, confusing, and misleading. Only a subset of very small proteins or peptides examined here translocated through the pores, others didn't. Reading the entire manuscript one is not left with the sense that a

"general rationale" is being presented. The authors either have to explain better what they mean, make more convincing arguments for this statement, or simply rewrite the concluding statement altogether.
All of the tested smaller proteins (11 kDa; EGF and b2-microglobulin) and peptides (Endothelin 1, 2 and angiotensin I) translocated through WtFraC, while one larger protein (chymotrypsin, 25 kDa) was shown not to translocate, presumably due to sterical reasons. Therefore, it is likely that all proteins with a diameter smaller than the diameter of the nanopore will translocate FraC nanopores, given the pH of the solution is tuned. However, we are aware that not all proteins translocate the nanopore, hence the use of 'entry' throughout the manuscript.
Maybe from the abstract and title the reviewer expected a manuscript solely on protein sequencing with nanopores. Indeed, one of the aims of this work is assessing FraC nanopores as sensors for protein sequencing, the other is the discrimination of biomarker irrespective of translocation. We have now made this point more clearly by specifying that FraC is used for folded and unfolded protein analysis.
However, we presented a general rationale for the capture (and translocation) of small proteins and unfolded peptides by the electro-osmotic flow. Crucially, in the context of protein sequencing, we showed that the electro-osmotic flow can be used to capture and translocate of unfolded peptides, irrespectively of their charge, when the pH of the solution is lowered to 4.5 . This is an important, because it is now possible to transport unfolded protein across a nanopore without using enzymes.

We have further clarified these points in the main text

The sentence ending in line 51 needs a reference(s).
We did not find very suitable references for this. However, we think it is obvious that if the analyte molecule has no charge, the electric field does not have an influence the transport dynamics across the nanopore. This is because the electrophoretic force, Fep, depends on the charge of the molecule as Fep=qE (q is the charge and E the electric field). Thus, for analytes with zero charge, the Fep is zero.

Line 62 to 64: It appears that if the authors would have chosen a slightly more acidic pH, such as pH 4.0, the condition that they say can not be achieved might actually have been achieved -- unless the authors know that at pH 4.0 the direction of WtFraC is reversed compared to pH 4.5.
We have tried lower pHs and we observed the capture of Endothelin 1 at -50 mV (analyte added to the cis solution), albeit the current blockades were sometimes 'nosier' than at higher pH values. We settled at pH 4.5 because is the closest to physiological pH, which is important if enzymes will be added to the system to control the transport of unfolded polypeptides across the nanopore.

For a high profile journal such as Nature Communications, the "writing" of this manuscript could be improved, both on the sentence level and the "storytelling" level. Early in the results section the text is hard to read because it presents detail that appears at times redundant with the figures or could possibly be put better in context rather than stating quantitative values (for instance in lines 124 to 136 as well as on the previous page). For this reason, the manuscript seems to lose the plot at times.
We amended our manuscript. Further, in order to comply with Nature Communications style, we have reorganized the results and discussion sections which at the same time improve the readability of the manuscript.

In both the introduction and the discussion, there seems to be a fixation on protein sequencing that doesn't seem particularly relevant, given that this paper makes no effort to sequence the analytes. Possibly move the speculation about FraC possible usefulness for protein sequencing towards the end of the discussion and discuss better why the authors feel that it may be an excellent pore for that (which it may well be but which will have to be demonstrated).
We have followed the suggestion of the reviewer and moved the discussion of protein sequencing towards the end of the manuscript. However, we still feel this manuscript represent an important step to understand whether nanopore protein sequencing is possible. In fact, the thorough understanding of conditions enabling the capture and translocation of peptides (or proteins) is of paramount importance for protein sequencing with any pore (if polypeptides cannot be captured and translocated there cannot be protein sequencing). Our finding about the electro-osmotic capture of different polypeptides, which is important in this context because it suggest that the EOF might be able to provide enough force to the polypeptides inside the nanopore despite the charge of the amino acid sequence.

The authors should discuss whether the concentrations of biomarkers that they demonstrated for their experiments are in the relevant physiological and pathophysiological range for these biomarkers. The authors should also touch on possible anticipated challenges when applying this approach to biomarker detection in relevant biofluids.
We have added a paragraph in the main text (discussion) describing this.

Line 126: What do the authors mean by "...the blockades...became homogeneous..."?
We have specified that the homogeneity refers to the residual current level.

The large body of interesting results presented in Figure 3 should be discussed in more detail. For instance, the results on kon rates for different peptides are scarcely discussed and put into context. What is their relevance?
We add a discussion about the kon dependence on voltage in results and the discussion session. However, we avoided an in-depth biophysical characterization of the data because we feel it is beyond the scope of this work. We performed this

experiment to establish the sensitivity of our method. A linear dependency of the capture frequency with the applied potential (chymotrypsin and β2-microglobulin) suggest that the capture process is diffusion-limited. However, we recorded more complex voltage dependencies for EGF and Endothelin 1, which might be related to the slightly negative overall charge of the polypeptide at pH 4.5. For negatively charged molecules the increase of electro-osmotic flow is partially balanced by the increased electrophoretic force that opposes translocation.

The "reversal potential" label above Fig. 2b is confusing, as "reversal potential" refers only to the x-intercept of those curves.
We agree with the reviewer and we have removed the label from the figure.

Just a suggestion: Wouldn't the graphs in Fig. 3 better convey the point that dwell time, Ires%, and kon are potential fingerprinting parameters if they all had the same axes, even if it's worse from a data presentation perspective?
We believe we have to compromise to display also subtle details. For example for β2-microglobulin, EGF and endothelin 1 we show the heatplot using identical axes. On the other hand, the bimodal behavior of EGF dwell times would be not visible on a y-axis of chymotrypsin for example. Hence, we prefer to keep the current arrangement.

First sentence in the conclusion: Mention that detection of single biomarkers in pure buffer solutions is possible and shows differences between peptides and in a binary mixture entdothelin I and II could be distinguished rather than a general statement that may imply that these biomarkers may be distinguishable in a mixture of all of them (or leave the sentence as is and show this capability).
We left the sentence as it is. We added a new figure (New Figure 4 in main text) from a new experiment showing discrimination of β2-microglobulin, EGF and endothelin 1 in a mixture.

The last sentence in the conclusion appears too strong "...it is likely...". Consider, "...it may be possible..."
We changed this.

REVIEWERS' COMMENTS:

Reviewer #1 (Remarks to the Author):

The authors answer well to the questions, comments and recommendations.
My recommendation is to accept this revised manuscript version for publication in Nature Communications
congretulations for this very interessting work

Reviewer #2 (Remarks to the Author):

This high quality work from Dr. Maglia’s group has introduced a new protein nanopore FraC for the proteomic characterization of the small proteins and peptides. FraC has shown remarkable discrimination ability to resolve small peptides with only one amino acid variation. The conclusion is backed up by solid experimental results and the manuscript is written very clearly. The revised document has corrected the errors in the previous version, included comprehensive coverage on the topic of nanopore protein analysis in the introduction. The revision has improved the presentation of the paper. I recommend the manuscript to be published in Nature Communication.

Reviewer #3 (Remarks to the Author):

The authors addressed most of my comments and the manuscript now reads very well. As stated before, the findings are very interesting and the data is convincing and presented clearly.

Only very minor comments remain, which do not have to be addressed and are probably matters of opinion:

I now see why the authors are stating that ET-1 differs only by one amino acid from ET-2, although I would argue that the position of an amino acid in the primary sequence of a peptide can be as important and influential as the nature of this amino acid (to illustrate this point imagine two peptides, in which the only difference between them would be that two amino acids would have simply swapped positions -- according the authors one could then say that these two peptides would not differ in amino acids. While this may be true, I think it is misleading since these two peptides could have different biological function). Therefore, in my mind ET-1 differs by two amino acids from ET-2 (one amino acid changed position and another one is different). This is may be a matter of opinion and Fig 5b does makes it clear....I am just not sure if I would stress, as much as the authors do, that these two peptides differ by only one amino acid -- it took my a while to understand why its not two.

L342: I am not sure that EOF is the only option to move proteins through the pore....the authors themselves state that enzymes might push or pull peptides or proteins through the pore and there could be other mechanisms.

L 38: I still think is it a long way to reliable amino acid analysis of folded and unfolded proteins with nanopores and to me the "rationale" is not so clear yet. I would formulate more carefully but with the changes in the discussion, the reader can now get a good idea what is and is not currently possible.

Overall this is very good work and I enjoyed reading and reviewing it.

REVIEWERS' COMMENTS:

Reviewer #1 (Remarks to the Author):

The authors answer well to the questions, comments and recommendations.
My recommendation is to accept this revised manuscript version for publication in Nature Communications congretulations for this very interessting work

Reviewer #2 (Remarks to the Author):

This high quality work from Dr. Maglia's group has introduced a new protein nanopore FraC for the proteomic characterization of the small proteins and peptides. FraC has shown remarkable discrimination ability to resolve small peptides with only one amino acid variation. The conclusion is backed up by solid experimental results and the manuscript is written very clearly. The revised document has corrected the errors in the previous version, included comprehensive coverage on the topic of nanopore protein analysis in the introduction. The revision has improved the presentation of the paper. I recommend the manuscript to be published in Nature Communication.

Reviewer #3 (Remarks to the Author):

The authors addressed most of my comments and the manuscript now reads very well. As stated before, the findings are very interesting and the data is convincing and presented clearly.

Only very minor comments remain, which do not have to be addressed and are probably matters of opinion:

I now see why the authors are stating that ET-1 differs only by one amino acid from ET-2, although I would argue that the position of an amino acid in the primary sequence of a peptide can be as important and influential as the nature of this amino acid (to illustrate this point imagine two peptides, in which the only difference between them would be that two amino acids would have simply swapped positions -- according the authors one could then say that these two peptides would not differ in amino acids. While this may be true, I think it is misleading since these two peptides could have different biological function). Therefore, in my mind ET-1 differs by two amino acids from ET-2 (one amino acid changed position and another one is different). This is may be a matter of opinion and Fig 5b does makes it clear....I am just not sure if I would stress, as much as the authors do, that these two peptides differ by only one amino acid -- it took my a while to understand why its not two.

We see why our statement might confuse the reviewer. However, the substitution can be rationalized as: methionine 6 in ED-1 is replaced by a tryptophan and moved to position 5 in ED-2. Nonetheless, we acknowledge the concern of the reviewer and we added a sentence in the main text to describe this.

L342: I am not sure that EOF is the only option to move proteins through the pore....the authors themselves state that enzymes might push or pull peptides or proteins through the pore and there could be other mechanisms.

We agree with the reviewer that the EOF is one of the options to control the transport of polypeptides across nanopores. However, we believe it is by far the best option. This is because if enzymes are used, then the nanopore should be used as a physical barrier to unfold the protein, which would pose many engineering challenges. On the other hand, if the EOF can be made to effectively transport unfolded polypeptides, then the unfolding can be obtained outside the nanopore by a variety of other means (chaotropic agents, temperature, enzymes, etc).

L 38: I still think is it a long way to reliable amino acid analysis of folded and unfolded proteins with nanopores and to me the "rationale" is not so clear yet. I would formulate more carefully but with the changes in the discussion, the reader can now get a good idea what is and is not currently possible.
We agree that several technical problems still need to be addressed in order to fulfill nanopore sequencing or proteomics. Here we focused in the key issues of capture, translocation and recognition. We are glad the reviewer appreciated our effort in the discussion to address these challenges.

Overall this is very good work and I enjoyed reading and reviewing it.
